# Extreme sensitivity of gene expression in human SH-SY5Y neurocytes to ultra-low doses of *Gelsemium sempervirens*

**DOI:** 10.1186/1472-6882-14-104

**Published:** 2014-03-19

**Authors:** Marta Marzotto, Debora Olioso, Maurizio Brizzi, Paola Tononi, Mirco Cristofoletti, Paolo Bellavite

**Affiliations:** 1 Department of Pathology and Diagnostics, University of Verona, Strada Le Grazie 8, Verona 37134, Italy; 2 Department of Statistical Sciences, University of Bologna, Via delle Belle Arti 41, Bologna 40126, Italy; 3 Department of Biotechnology, University of Verona, Strada Le Grazie 15, Verona 37134, Italy

## Abstract

**Background:**

*Gelsemium sempervirens* L. (*Gelsemium s*.) is a traditional medicinal plant, employed as an anxiolytic at ultra-low doses and animal models recently confirmed this activity. However the mechanisms by which it might operate on the nervous system are largely unknown. This work investigates the gene expression of a human neurocyte cell line treated with increasing dilutions of *Gelsemium s.* extract.

**Methods:**

Starting from the crude extract, six 100 × (centesimal, c) dilutions of *Gelsemium s.* (2c, 3c, 4c, 5c, 9c and 30c) were prepared according to the French homeopathic pharmacopoeia. Human SH-SY5Y neuroblastoma cells were exposed for 24 h to test dilutions, and their transcriptome compared by microarray to that of cells treated with control vehicle solutions.

**Results:**

Exposure to the *Gelsemium s.* 2c dilution (the highest dose employed, corresponding to a gelsemine concentration of 6.5 × 10^-9^ M) significantly changed the expression of 56 genes, of which 49 were down-regulated and 7 were overexpressed. Several of the down-regulated genes belonged to G-protein coupled receptor signaling pathways, calcium homeostasis, inflammatory response and neuropeptide receptors. Fisher exact test, applied to the group of 49 genes down-regulated by *Gelsemium s.* 2c, showed that the direction of effects was significantly maintained across the treatment with high homeopathic dilutions, even though the size of the differences was distributed in a small range.

**Conclusions:**

The study shows that *Gelsemium s*., a medicinal plant used in traditional remedies and homeopathy, modulates a series of genes involved in neuronal function. A small, but statistically significant, response was detected even to very low doses/high dilutions (up to 30c), indicating that the human neurocyte genome is extremely sensitive to this regulation.

## Background

*Gelsemium sempervirens* (*Gelsemium s.*), also called yellow jasmine, is a plant belonging to the Loganiaceae family. All parts of the plant contain the major active principle gelsemine as well as other toxic strychnine-related alkaloids, such as gelseminine and sempervirine [1-3]. In the phytotherapy literature, *Gelsemium s.* has been reported to show sedative, analgesic and anti-seizure properties [4,5] while in the homeopathic Materia Medica and literature, *Gelsemium s.* is described as a remedy for a variety of anxiety-like psychological and behavioral symptoms [6-9]. The anxiolytic, antidepressant and/or analgesic action of *Gelsemium s.* extracts and its purified components has been recently demonstrated in animal models [10-16]. Other reports in the literature suggest this plant species may exhibit anticancer and immune-modulating activity [17-20].

The question of dosage is obviously central to pharmacology and of particular interest in homeopathic pharmacopoeia, where the procedure of serial dilutions followed by shaking has sparked much debate. The original extract (Mother Tincture, MT) is generally obtained by grinding the medicinal plant matter with a mortar and pestle and dissolving it in ethanolic solution. According to the most widely-used French pharmacopoeia, the first centesimal (1c) dilution is obtained by dissolving one volume of MT in 99 volumes of 30% ethanol in water and then subjecting it to vigorous shaking (succussion or “dynamization”). Subsequent c dilutions are prepared by repeating the same procedure. Although the lower dilutions (i.e., 2c to 5c) contain substantial amount of the original active phytochemical substances, their concentration progressively decreases as the number of dilutions increases. Thus, in order to address possible mechanisms of action of high dilutions, physical or chemical mechanisms involving changes imparted to the solvent itself have been hypothesized [21-23]. This is a fairly controversial question in the literature on *Gelsemium s.,* since most authors have investigated only a narrow range of doses or dilutions. It is also important, when dealing with elusive phenomena such as biological responses to diluted and dynamized substances, to take special care with the controls: the recent consensus recommendation among researchers in this field is for protocols that use the diluted and succussed vehicle solution as a control, however this is still a debated theme and has been done only in few cases [24,25].

Previous investigations in our laboratory [26,27] have shown a significant anxiolytic-like activity of *Gelsemium s.* high dilutions (namely 5c, 7c, 9c and 30c according to different test paradigms) in mice, using emotional response models. Other laboratories have also reported *in vivo*[16,19,28,29] or *in vitro*[30] effects of *Gelsemium s.* in extremely low doses or high dilution/dynamization, but its action at the cellular level has not been fully clarified. To follow up the above evidence of an anxiolytic effect in animal models, we decided to investigate the *Gelsemium s.* mechanism of action in neuronal models by assessing the drug effects on whole genome expression changes. The SH-SY5Y and IMR-32 human neuroblastoma cells were used since are widely employed in neuropharmacology [31-33]. Finally, this approach allowed us to test several replications of multiple doses and dilutions of the remedy, taking advantage of high-throughput and easily reproducible microarray technology.

Cells were treated with a wide variety of doses: in total, we tested 6 increasing dilutions - which was the maximum sample size permitted by technical constraints - from the low dilution 2c (dilution factor 10^4^) to the extremely high dilution 30c (dilution factor 10^60^). The 5c, 9c, 30c dilutions are among the most frequently used drug formulations in complementary therapies on humans [34]. The drug effects were compared with those of the same solvent used for the dilutions of *Gelsemium s.*, just without the plant extract (control solutions). After testing for possible toxic effects of any dilution on cell viability, their effectiveness in changing gene expression was evaluated using a microarray designed for the whole human transcriptome. *Gelsemium s.* 2c was checked in SH-SY5Y and IMR-32 cells and the most responsive cell line was chosen for testing also higher dilutions/dynamizations.

## Methods

### Preparation of *Gelsemium s*. and control solutions

The homeopathic dilutions/dynamizations were prepared in a manner comparable to methods used by commercial manufacturers, i.e. using 30% ethanol for all dilution/succussion steps. Since ethanol at higher concentration may be toxic for cells the 100x, last dilution/succussion was made in pure water. The detailed procedure was carefully repeated in all experiments and precisely reported below, since it is relevant as basic science research on homeopathic medicine progresses. Whole hydroalcoholic extract (MT) of *Gelsemium s.* was produced by Boiron Laboratoires, Lyon (F) according to the French Homeopathic Pharmacopoeia [35]. The gelsemine content in the MT was 6.5 × 10^-4^ M. MT was diluted 100 times in 30% ethanol/distilled water to obtain the 1c dilution. Subsequent serial 100× dilutions up to 29c, each followed by vigorous succussion (shaking) were then prepared in the same solvent using glass bottles. 30-ml bottles containing 1c, 2c, 3c, 4c, 8c and 29c dilutions were supplied by the manufacturer wrapped in aluminum foil and stored in the dark at room temperature in a metal cupboard. The control solutions (solvent) were prepared as the drug dilutions just without the plant extract. The 1c, 2c, 3c, 4c, 8c and 29c solvent samples contain only 30% ethanol/distilled water, but differ for the number of succussions performed. To prepare the final dilutions used in the tests, immediately before the experiments, 0.05 ml of the solutions (*Gelsemium s.* and controls) were added to 4.95 ml of distilled sterile-filtered water (Sigma-Aldrich) in a sterile 15 ml Falcon polystyrene plastic tube and shaken in a DinaA mechanical shaker for 7.5 sec (150 strokes). This yielded the 2c, 3c, 4c, 5c, 9c and 30c succussed dilutions, with ethanol concentration lowered to 0.3% (v/v) (final 0.03% in the assay system).

UV-visible absorption spectra of *Gelsemium s.* samples were performed with a Jasco V550 double-beam spectrophotometer using quartz cuvettes with 1-cm optical path and control solutions as the reference samples.

### Exposure of cells to *Gelsemium s*. and control dilutions

Human neuroblastoma cell line SH-SY5Y [36,37], kindly provided by prof. Ubaldo Armato (Department of Life and Reproduction Sciences, University of Verona), was grown in DMEM-F12 (1:1) medium (Lonza, Walkersville, MD, USA), supplemented with 10% foetal bovine serum (FBS; Lonza), penicillin (100 units ml^-1^) and streptomycin (100 mg ml^-1^) (Lonza). The culture medium was replaced every three days. The cells were grown in Greiner plastic culture flasks at 37°C in a 5% CO_2_ atmosphere, until 80% confluence was reached. Cells were propagated after reactivation of cryogenates until the fourth culture passage and then used for the gene expression assay. Cells were counted in duplicate in a Thoma counting chamber after staining with Turk blue reagent. For the analysis of differential gene expression, SH-SY5Y cells were plated onto Petri dishes (Ø 100 mm) and, the day after this plating, the culture medium was replaced with the same medium (10 ml) supplemented with 2% FBS. After 24 h, 1 ml of *Gelsemium s.* or control dilution was added to the cell culture and maintained at 37°C with 5% CO_2_ in a humidified atmosphere (90% humidity) for a further 24 h. Four replicate experiments were carried out under identical conditions. In three experiments, *Gelsemium s.* 2c and the respective control were tested on IMR-32 neuroblastoma cell line (CCL-127 purchased from ATCC, Manassas, VA, USA), grown and treated under the same conditions, except that EMEM medium (Lonza) was used instead of DMEM-F12.

### Cell viability assay

The cytotoxic action of the *Gelsemium s.* or ethanol dilutions on SH-SY5Y cells was assessed by the WST-1 assay [38]. In this test, cell viability is reflected by mitochondrial dehydrogenase activity in cleaving tetrazolium salts (WST-1 reagent, Roche Molecular Biochemicals -Mannheim, Germany) to soluble formazan. A total of 20,000 cells per well were seeded in a 96-well microplate in the DMEM-F12 medium with 10% FBS and left to adhere for 16 h. Then the culture medium was replaced with 200 μl of the same medium supplemented with 2% FBS. Drug and control solutions (22 μl) were then added (6 replicates of each condition for each plate) and the plate was incubated at 37°C in a 5% CO_2_ atmosphere. After 24 h, 1:10 (v/v) pre-warmed WST-1 solution was added to the cells and the plate incubated for 3 h. The absorbance (OD) of the samples was measured using a Victor3 multilabel reader (PerkinElmer, Shelton, CT, USA ) at 450 nm, and cell metabolic activity was evaluated as the difference between OD at 3 h and OD at T0.

### Measurement of intracellular Ca^2+^ concentration

Increase in intracellular Ca^2+^ was monitored as described [39] with minor modifications. SH-SY5Y cells were inoculated in 96-well black microplates (flat transparent bottom) with a density of 80,000 cells/well and left to adhere for 16 h. The culture medium was removed from the wells, and the cells were washed with warm Hank’s basal saline solution (HBSS, Sigma-Aldrich) with 20 mM Hepes (Sigma-Aldrich) and incubated with loading medium (100 μl/well) at 37°C for 40 min in the dark, with 5% CO_2_ in a humidified atmosphere. The loading medium was made up of the Ca^2+^-sensitive dye Fluo-4 AM (4.5 μM) (Invitrogen, Paisley, UK) and probenecid (2.5 mM) (Invitrogen) in HBSS. After incubation, the cells were washed and incubated with warm HBSS containing 2.5 mM probenecid at 37°C for 30 min in the dark. At the indicated time, carbachol (Sigma-Aldrich) was added at the final concentrations of 1, 5, 10, 20 μM and the plate was transferred to a Victor3 multilabel reader (PerkinElmer) for the measurements. Each dose was measured in triplicate and compared with a blank for about 15 min using the kinetic mode.

### RNA isolation and quality controls

Cells exposed to 24 h *Gelsemium s.* or control solutions were harvested with trypsin-EDTA-PBS treatment (5 mg L^-1^, Lonza) and counted. Then, total RNA was promptly extracted (from 3.5 × 10^6^ cells) using the Qiagen RNAeasy Mini Kit (Qiagen GmbH, Hilden, Germany) following the manufacturer’s instructions (Animal cells Spin protocol), including genomic DNA elimination step in column. RNA extraction was performed within 20 min from cell detachment. The RNA samples were concentrated by precipitation with 2.5 volumes of ice-cold absolute ethanol in presence of 0.3 M Na acetate. RNA yield was determined by a NanoDrop 1000 spectrophotometer (Thermo Scientific, Wilmington, DE, USA) and RNA integrity was then evaluated using the 2100 Bioanalyzer (Agilent, Santa Clara, CA, USA).

### cDNA synthesis, labelling and microarray hybridisation

Microarray analysis was performed on a 12 × 135 K (i.e. made with 12 sub-arrays and 135,000 probes per sub-array) human NimbleGen microarray chip (Roche NimbleGen, Madison, WI, USA, catalogue no. 05 543 789 001, design 100718_HG18_opt_expr_HX12) containing 45033 genes with 3 probes per target gene. The microarray is based on HG18, Build 36; cDNA synthesis, labelling and hybridization were performed according to manufacturer’s protocols (http://www.nimblegen.com/support/dna-microarray-support.html; see file 05434505001_NG_Expression_UGuide_v6p0.pdf). Briefly, 10 μg total RNA for each sample was used to synthesize cDNA using a SuperScript double-strandedcDNA synthesis kit (Invitrogen) with oligo(dT) primers for amplification. After further evaluation of integrity and yield with the Bioanalyzer, the cDNA samples were labelled with Cy3 using a NimbleGen One-Color DNA labelling kit (Roche). 4 μg of Cy3-cDNA were hybridized on each subarray for 16 h at 42°C. All 12 samples (6 *Gelsemium s.* and 6 controls) for each experiment were hybridized in the same chip and processed simultaneously. Sample tracking controls were used to ensure against cross contaminations or erroneous loading in the array. The procedure was repeated for four and three biological replicates with SH-SY5Y and IMR-32 cells, respectively.

The arrays were scanned with a GenePix 4400A scanner (Molecular Devices Corp., Sunnyvale, CA, USA) and scanned images (TIFF format) were then imported into the NimbleScan software for grid alignment and expression data analysis. Quality control of the array images was performed on the basis of the parameters reported in the Experimental Metrics Report as indicated by the NimbleGen Software Guide v3.0. The parameters assessed the absence of spatial biases of the fluorescence within each subarray, the homogeneity of the mean signal among the subarrays and the acceptable level of background (empty and random spots) before background correction and intra-array normalization. Gene calls were generated using the Robust Multichip Average (RMA) algorithm as described by Irizarry et al. [39]. Normalization was performed using quantile normalization as described by Bolstad et al. [40]. The data have been deposited in NCBI's Gene Expression Omnibus [41] and are accessible through GEO Series accession number GSE42236.

### Real time quantitative RT-PCR

A qRT-PCR analysis was performed on SH-SY5Y neuroblastoma treated with *Gelsemium s*. 2c or the control 2c, to verify the gene expression profile of AIPL1, ALPK3, BIRC8, C1ORF167, DDl1, EN2, GALR2, GPR25, LST1, OR4X1, OR5C1, KLKBL4 and TAC4 genes, that were identified by microarray analysis. UPL hydrolysis probes and primers (RealTime ready Assays, Roche) were specifically designed and experimentally validated to match the differentially expressed transcript Id identified by Nimblegen microarray. One μg of RNA previously extracted (Qiagen), quantified spectrophotometrically (Nanodrop) and further DNase treated (Turbo DNA-free kit, Ambion), was reverse transcribed using Transcriptor First Strand Synthesis kit with oligo dT (Roche) and subsequently 250 ng of cDNA were pre-amplified with a pool of primers following the instruction of RealTime Ready cDNA Pre-Amp Master kit (Roche). The pool consisted of the RealTime Ready Assays primers specific for genes listed above diluted 1:10 each in water PCR-grade. One to 20 diluted pre-amplified cDNA was put in qPCR with the gene specific RealTime Ready Assays and with FastStart Universal Probe Master-Rox (Roche). Briefly, the reaction mixture consisted of 10 μl of 2X FastStart Universal Probe Master-Rox, 1 μl of 20X RealTime ready Assay, 1 μl of template cDNA diluted 1:10 and nuclease free water up to 20 μl. Three different technical replicates were analyzed for each cDNA sample in the same assay and β–actin (ACTB gene ID: 60) and β-2 microglobulin (B2M gene ID: 567) were used as housekeeping genes for the normalization. The StepOne Plus Real-time PCR System (Applied Biosystem, USA) was used to monitor the hydrolysis probe signal generated with a standard thermal profile specific for this kind of probe, i.e.10 min of 95°C, followed by 40 cycles of 95°C 15 sec, 1 min of 60°C. The quantification cycle (Cq) was determined by using the log view of the ΔRn amplification plots, normalized by the internal ROX reference dye, whereas the relative fold change (FC) in the expression levels was determined with the ΔΔCq method, taking the mean of the three PCR replicates. Data are presented as Log2 transformation of FC.

### Statistics

The experimental model had dose–response setup, including 6 dilutions of *Gelsemium s*. and 6 corresponding controls. The main working variable was the Log2-transformed fluorescence value of microarray analysis of gene expression. Data from 4 independent experiments were considered. Expecting effects to diminish with increasing dilution, we focused to a pair-wise comparison between *Gelsemium s.* dilutions and the vehicle controls instead of an overall comparison analysis. Two consecutive statistical approaches were followed. The first approach analyzed the complete transcriptome dataset and was aimed to select the DEGs that were most significantly affected by treatment at the highest dose; linear model (Limma) was applied to compare the expression values from *Gelsemium* 2c treated and the mean of controls (see details below). The second approach analyzed only the expression values of the selected DEGs when treated with highly diluted drugs or the corresponding controls. The main focus was to verify the null hypothesis that the higher *Gelsemium s.* dilutions did not affect the expression of the genes compared to control. For this analysis we used Friedman test as nonparametric ANOVA and Fisher’s exact test (see details below). The tests analyzed the distributions of the fold changes in the down- or up-regulated DEGs and determined whether the direction of effect for the DEGs detected in the 2c concentration was maintained across all other dilutions (3c-30c).

In the first part of analysis, a linear modelling approach and the empirical Bayes statistics as implemented in the Limma package [42] were employed for differential expression analysis. The Limma test was applied to compare *Gelsemium s.* dilutions with controls, or controls between each other. The p-values were adjusted for the False Discovery Rate (FDR) on the 45033 cases using the Benjamini and Hochberg method [43]. No pre-filtering to the dataset (variant-based or minimal expression-based) was applied to avoid a-priori loss of results when studying minimal doses of drug. Log2 fold change was calculated as Log2-transformed fluorescence value of *Gelsemium s.* dilutions minus Log2-transformed fluorescence value of mean of controls. DEGs were selected as significant and interesting for further analysis if their absolute value of Log2 fold change (|log2 fold change|) was higher than 0.5 and the adjusted *p*-value was <0.05.

In the second part of the analysis, the significant DEGs in 2c treatment were divided in two groups (considered as gene-sets) according to their direction of change, including down- and up-regulated genes; data referred to the same dilution (from 2c to 30c of both treatments and respective controls) were joined, treating the single gene as a statistical unit and the mean of four replications as the corresponding datum. Statistical significance of the overall differences between expression profiles of gene-sets (down- and up-regulated) in various treatment conditions was calculated by the Friedman multi-sample test using SPSS software, version 17 (SPSS Inc., Chicago, IL, USA). The Friedman test is a nonparametric test for multiple related samples (in our case, the expression level of multiple matched samples from cells treated with 6 *Gelsemium s.* dilutions or 6 control solutions) that checks the null hypothesis that multiple ordinal responses come from the same population. Following a significant result of Friedman test, frequency of down-regulated vs upregulated genes were calculated; |log2 fold change| lower than or equal to 0.05 were considered to be null. The significance of distributions for each dilution was analyzed by the Fisher’s exact test, which calculates the exact probability of getting, only by chance, the observed values or more extreme ones. A randomly selected set of 49 genes, for comparison of frequency of down-regulated vs up-regulated genes with the *Gelsemium s.-*specific gene-set, was generated from the whole microarray dataset, using the specific function of the SPSS 17 software.

Gene expression profiles were clustered by the k-means clustering method and Pearson correlation metrics using the MeV 4.8.1 software (http://www.tm4.org/mev.html). The application “Figure of merit” (FOM) was used to set the number of clusters that best fit the dataset variability [44]. The FOM measures the average intra-cluster variance of the observations, estimating the mean error using predictions based on the cluster averages [45]. Gene functional classification and enrichment analysis were performed by DAVID Bioinformatics Resources 6.7 (http://david.abcc.ncifcrf.gov) [46]. Results of viability assay were analyzed by ANOVA and t-test comparing data from each *Gelsemium s*. dilution (G2c, G3c, G4c, G5c, G9c and G30c) with the corresponding controls (n = 12 replicates for each group).

## Results

### Cell morphology and function

**Figure 1:**
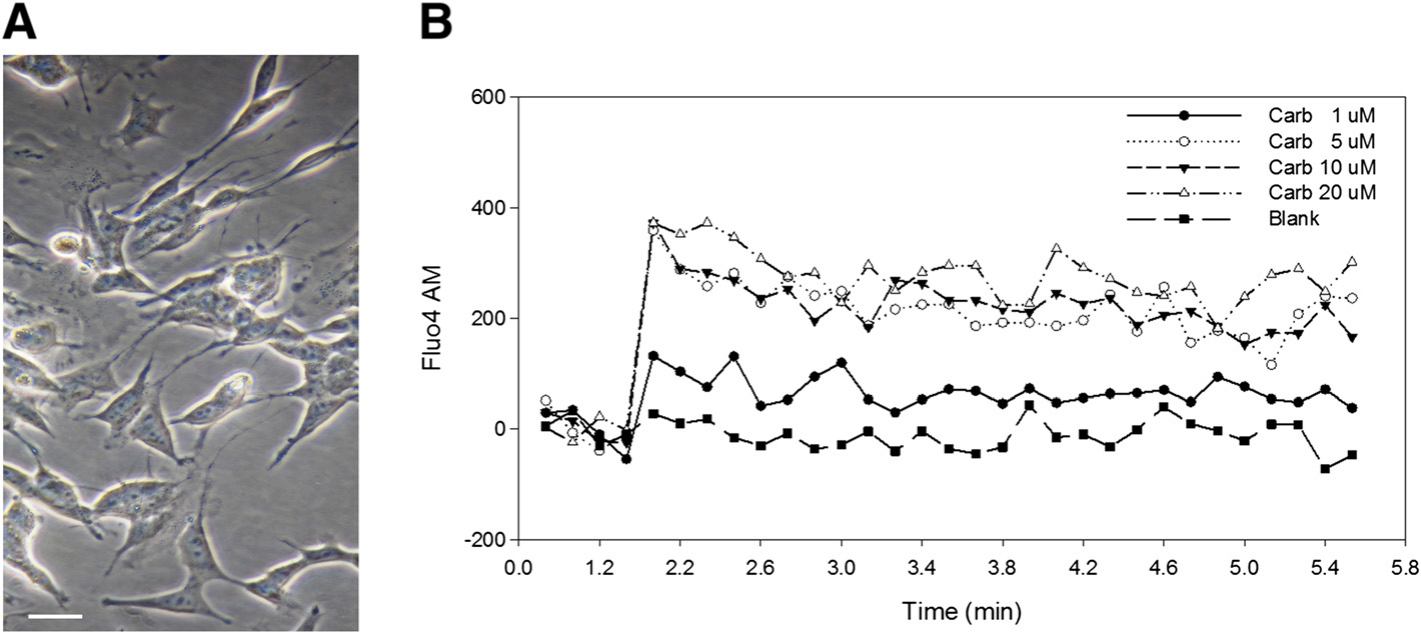
**Morphological and functional properties of SH-SY5Y neuroblastoma cells used in the assay. A**. Phase contrast micrograph of adherent cells cultured in Petri dishes. Bar, 10 μm. **B**. Spectrofluorometric measurement of intracellular calcium changes induced by carbachol at the specified doses.

The SH-SY5Y neuroblastoma cells used in the assay exhibited a neuron-like shape with visible axons and junctions when grown in Petri dishes (Figure 1A). To assess the basal neuronal reactivity of these cells, the culture was stimulated with different doses of the acetylcholine-analogue carbachol and the change in concentration of intracellular calcium was measured with the Ca^2+^-sensitive probe Fluo4-AM. As shown in Figure 1B, the cells were sensitive to the varying amounts of the neurotransmitter, and intracellular calcium concentration increased in a dose-dependent way.

### UV–VIS Spectra of *Gelsemium s.*

**Figure 2:**
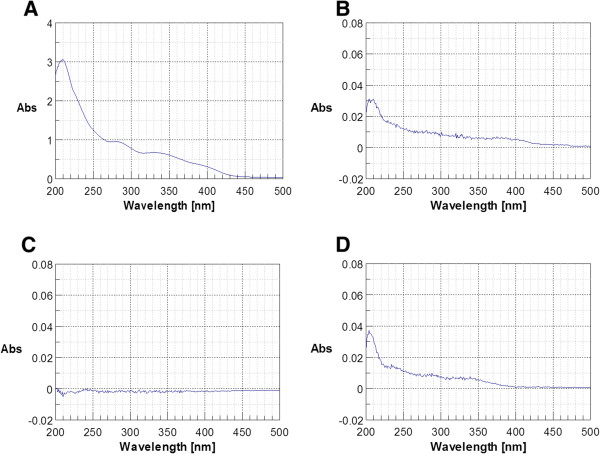
**UV–vis spectra of representative *Gelsemium s. *solutions. A**: *Gelsemium s.* 1c dilution supplied by the manufacturer, **B**: *Gelsemium s.* 2c dilution supplied, **C**: *Gelsemium s.* 3c dilution supplied, **D**: *Gelsemium s.* 2c dilution prepared by 100 × dilution of solution A and used in the experiments.

Figure 2 shows the absorption spectra of some of the preparations used in this study. The spectrum of the lowest dilution (1c) was considered as marker for the actual presence of plant extract. Spectra of the subsequent dilutions checked the effectiveness of the 100 × dilution steps, i.e. verified that a) the lower dilutions (from pharmaceutical factory and prepared in the laboratory) were comparable and b) the provided higher potencies were effectively diluted. The original 1c dilution supplied by the factory (panel A) was characterized by high absorption in UV region near 210 nm and by two absorption shoulders at 280 nm and 330 nm; no absorption in the visible spectrum region above 450 nm was detected. The original 2c dilution (panel B) showed a qualitatively similar spectrum, but with an absorption intensity about 100 times lower than that of 1c, indicating that the dilution was done correctly during the preparation process. The 3c dilution (panel C) has no significant absorption over the background noise level, which is as expected since it was produced by a 100 × dilution of 2c. The 2c solution prepared in the laboratory by a 100 × dilution of the original 1c in water (panel D) shows a spectrum with absorption features corresponding roughly to 1/100 of the spectrum of (panel A), indicating that the final dilution of the samples for use in cell assays was done correctly. The spectra of higher dilutions were below the detection limit for this technique (data not shown).

### Effect of *Gelsemium s*. dilutions on SH-SY5Y viability

**Figure 3:**
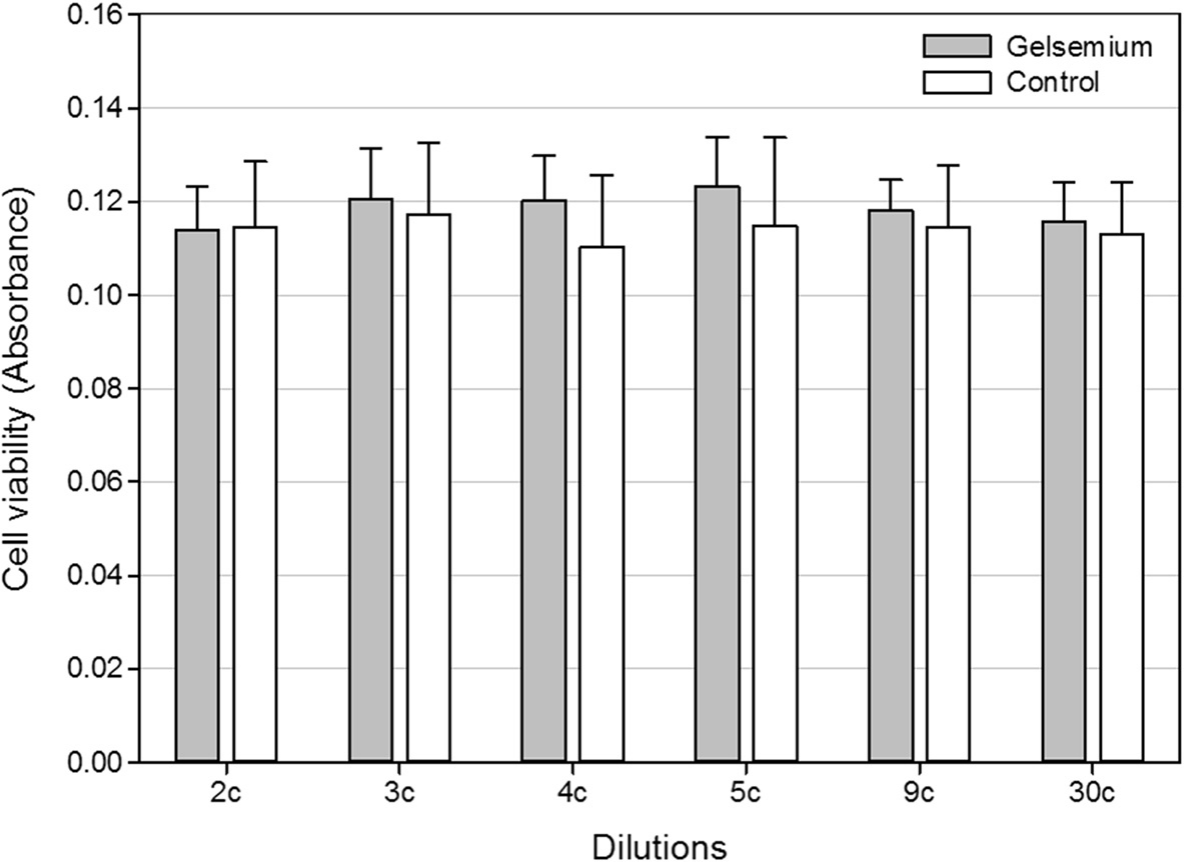
**Effects of *Gelsemium s. *on SH-SY5Y cell viability.** Cell viability was determined by WST assay after 24 h treatment with *Gelsemium s.* or Control dilutions. Values in abscissa are mean absorbance values ± SD (n = 6).

To evaluate whether the *Gelsemium s.* dilutions had any toxic effects, the viability of SH-SY5Y cells after exposure to drug dilutions for 24 h was checked by WST-1 spectrophotometric assay. As can be seen in Figure 3, *Gelsemium s.* dilutions did not impair cell viability as compared to controls. No significant differences in cell viability were observed between cells treated with the ethanol control solution 0.03% (v/v) and untreated cells (data not shown).

### Gene expression changes induced in SH-SY5Y cells

Global changes in gene expression produced by exposure to low doses or high dilutions of *Gelsemium s.* extracts in human SH-SY5Y neuroblastoma cells were investigated by microarray analysis, and the results selectively compared with the gene expression of cultures exposed to the vehicle solutions. Cells were incubated for 24 h with the 6 dilutions of *Gelsemium s.* or the corresponding controls, after which the 12 samples were rapidly processed and tested simultaneously on a 12 × 135 K NimbleGen chip. After running a total of 4 experiments, differential gene expression was analyzed. The general correlation values among the 48 normalized sub-arrays compared in the analysis (12 conditions and 4 replicates) was very high (Pearson correlation coefficient >97%, mean = 0.988), demonstrating the reproducibility of the experiments.

Preliminary Limma analysis, performed only with the control samples, excluded the presence of significant differences among the different diluted/succussed vehicles (adjusted *p > 0.05*), and authorized merging them in a unique control group. The difference in expression (Log2 fold change) between *Gelsemium s.* and the average of the controls was calculated for each dosage, and the results were compared to detect any trend in the response to increasing drug dilutions. In general, the range of changes in gene expression was quite narrow: out of a total of 45033 transcripts, in *Gelsemium s.* 2c and 3c, the lowest dilutions, only a small subset of genes (577 and 165, respectively) showed |log2 fold change| > 0.5. Among these DEGs, exposure to *Gelsemium s.* 2c promoted a statistically significant down-expression of 49 genes, while 7 genes were overexpressed (Limma analysis with adjusted *p < 0.05*) (Table 1). In general, mean fold changes in the mRNAs levels of cells treated with *Gelsemium s.* were small and only 4 genes showed |log2 fold change| > 0.8. No significant changes of housekeeping genes were recorded, as expected.

**Table 1:** **Differentially expressed genes after treatment with *Gelsemium s. *** **2c in SH-SY5Y cells**

**Gene ID**	**Transcript ID**	**Symbol**	**Log2 fold change**	***p***^**1**^	**Description**
7940	AF000424	LST1	-0.84 ± 0.14	0.04	Leukocyte specific transcript 1
390113	NM_001004726	OR4X1	-0.83 ± 0.06	0.01	Olfactory receptor, family 4, subfamily X, member 1
23746	AJ830742	AIPL1	-0.82 ± 0.16	0.04	Aryl hydrocarbon receptor interacting protein-like 1
284498	AL833920	C1orf167	-0.80 ± 0.17	0.05	Chromosome 1 open reading frame 167
221191	AK058068	Klkbl4	-0.79 ± 0.12	0.04	Plasma kallikrein-like protein 4
26658	NM_012377	OR7C2	-0.77 ± 0.07	0.01	Olfactory receptor, family 7, subfamily C, member 2
112401	BC039318	BIRC8	-0.76 ± 0.11	0.00	Baculoviral IAP repeat-containing 8
2848	NM_005298	GPR25	-0.75 ± 0.15	0.02	G protein-coupled receptor 25
55803	NM_018404	ADAP2	-0.75 ± 0.11	0.02	ArfGAP with dual PH domains 2
386676	NM_198690	KRTAP10-9	-0.73 ± 0.12	0.04	Keratin associated protein 10-9
4353	X04876	MPO	-0.72 ± 0.15	0.04	Myeloperoxidase
N/A	AY358413	N/A	-0.71 ± 0.18	0.02	Homo sapiens clone DNA59853 trypsin inhibitor
392391	NM_001001923	OR5C1	-0.71 ± 0.05	0.04	Olfactory receptor, family 5, subfamily C, member 1
N/A	AK094115	N/A	-0.70 ± 0.11	0.04	Homo sapiens cDNA FLJ36796 fis, clone ADRGL2006817
55287	BC020658	TMEM40	-0.70 ± 0.15	0.02	Transmembrane protein 40
54209	NM_018965	TREM2	-0.69 ± 0.10	0.02	Triggering receptor expressed on myeloid cells 2
150365	AK097834	RP5-821D11.2	-0.68 ± 0.17	0.02	Similar to mouse meiosis defective 1 gene
400934	NM_207478	FLJ44385	-0.68 ± 0.09	0.04	FLJ44385 protein
255061	NM_170685	TAC4	-0.67 ± 0.14	0.01	Tachykinin 4 (hemokinin)
644065	XM_931993	LOC644065	-0.65 ± 0.23	0.04	Hypothetical protein LOC644065
1339	NM_005205	COX6A2	-0.64 ± 0.17	0.01	Cytochrome c oxidase subunit VIa polypeptide 2
N/A	AK128093	N/A	-0.63 ± 0.09	0.04	Homo sapiens cDNA FLJ46214 fis, clone TESTI4012623.
53841	AY358368	CDHR5	-0.63 ± 0.11	0.04	Mucin-like protocadherin
9332	NM_004244	CD163	-0.63 ± 0.18	0.03	CD163 molecule
441239	XM_499305	LOC441239	-0.63 ± 0.22	0.05	Hypothetical gene supported by BC063653
7164	NM_001003397	TPD52L1	-0.62 ± 0.09	0.02	Tumor protein D52-like 1
11136	NM_014270	SLC7A9	-0.62 ± 0.09	0.04	Solute carrier family 7 member 9
389084	NM_206895	UNQ830	-0.62 ± 0.11	0.04	ASCL830
400224	XM_375090	FLJ44817	-0.62 ± 0.20	0.04	Similar to pleckstrin homology domain protein (5 V327)
647240	XM_934559	LOC647240	-0.60 ± 0.06	0.00	Hypothetical protein LOC647240
846	BC104999	CASR	-0.59 ± 0.06	0.00	Calcium-sensing receptor
116123	NM_138784	RP11-45 J16.2	-0.58 ± 0.09	0.04	Flavin-containing monooxygenase pseudogene
644280	XM_497769	LOC644280	-0.58 ± 0.06	0.05	Hypothetical protein LOC644280
57452	AB032956	GALNTL1	-0.57 ± 0.17	0.05	Alpha-D-galactosamine N-acetylgalactosaminyltransferase
414301	NM_001001711	DDI1	-0.56 ± 0.11	0.04	DDI1, DNA-damage inducible 1, homolog 1 (*S. cerevisiae*)
116535	BC016964	MRGPRF	-0.55 ± 0.17	0.01	MAS-related GPR, member F
8811	NM_003857	GALR2	-0.55 ± 0.07	0.04	Galanin receptor 2
10880	NM_006686	ACTL7B	-0.55 ± 0.12	0.04	Actin-like 7B
6368	NM_145898	CCL23	-0.55 ± 0.11	0.05	Chemokine (C-C motif) ligand 23
64581	BC071746	CLEC7A	-0.54 ± 0.08	0.04	C-type lectin domain family 7, member A
644003	XM_927256	LOC644003	-0.54 ± 0.11	0.04	Similar to Mucin-2 precursor (Intestinal mucin 2)
643514	XM_931594	LOC643514	-0.54 ± 0.10	0.03	Hypothetical protein LOC643514
374569	XM_935431	LOC374569	-0.54 ± 0.07	0.04	Similar to Lysophospholipase
84504	BC101635	NKX6-2	-0.53 ± 0.13	0.03	NK6 transcription factor related, locus 2 (Drosophila)
732	NM_000066	C8B	-0.53 ± 0.06	0.05	Complement component 8, beta polypeptide
146336	NM_182510	FLJ32252	-0.52 ± 0.03	0.01	Hypothetical protein FLJ32252
150763	BC042847	LOC150763	-0.51 ± 0.10	0.04	Hypothetical protein LOC150763
2020	NM_001427	EN2	-0.51 ± 0.08	0.04	Engrailed homolog 2
646258	XM_929203	LOC646258	-0.51 ± 0.11	0.04	Hypothetical protein LOC646258
154872	NM_001024603	LOC154872	0.51 ± 0.10	0.03	Hypothetical LOC154872
400866	NM_001001789	C21orf24	0.52 ± 0.12	0.05	Chromosome 21 open reading frame 24
9457	NM_020482	FHL5	0.55 ± 0.19	0.04	Four and a half LIM domains 5
55816	NM_018431	DOK5	0.56 ± 0.04	0.03	Docking protein 5
1446	NM_001890	CSN1S1	0.57 ± 0.09	0.04	Casein alpha s1
285600	AK130941	KIAA0825	0.63 ± 0.06	0.01	KIAA0825 protein
57538	NM_020778	ALPK3	0.76 ± 0.10	0.01	Alpha-kinase 3

The table includes the genes with absolute Log2 fold change higher than 0.5. Each gene is described via GeneBank accession number (Gene ID), Gene symbol (Symbol), NimbleGen array transcript designation (Transcript ID). The log2 fold change of expression compared to mean control vehicle-treated cells is displayed as mean ± SEM (n = 4 replicate experiments). ^1^adjusted *p* value with Benjamini-Hochberg correction, obtained by Limma statistical test.

### Gene expression changes induced in IMR-32 cells

**Figure 4:**
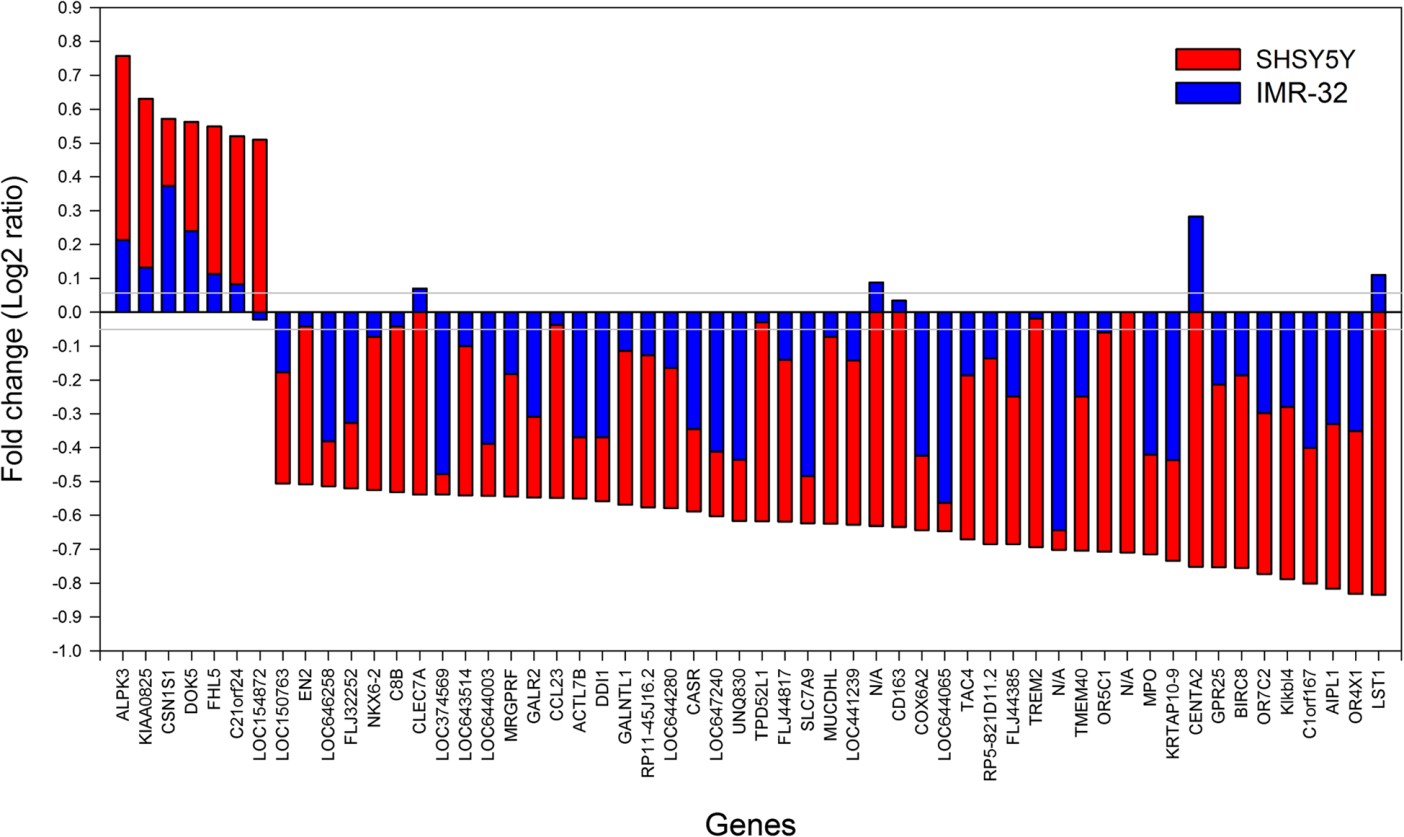
**Differential effect of *Gelsemium s. *on two cell lines.** Fold changes of the 56 selected genes in SH-SY5Y (red bars) and IMR32 (blue bars) cells after 24 h treatment with *Gelsemium s.* 2c.

To verify whether the effect of *Gelsemium s.* could be reproducible in different types of neurocytes, the *Gelsemium s.* 2c treatment and the corresponding ethanol controls were applied to the IMR-32 human neuroblastoma cell line. After three replicate experiments, the analysis did not detect significant changes between *Gelsemium s.* and controls if the cut-off values of |log2 fold change| > 0.5 and adjusted *p < 0.05* (Limma analysis with Benjamini and Hochberg correction) were applied. As observed in the SH-SY5Y cells, the global gene expression change in the IMR-32 cells was slight, since only 116 genes (0.25% of the transcripts) registered |log2 fold change| >0.5 (compared to the 577 genes in SH-SY5Y cells, corresponding to 1.3% of total). In any case, as shown in Figure 4, the changes in the 56 selected genes of SH-SY5Y cells were in the same direction in the IMR32 cells. In fact, 44 of the 49 genes that were down-regulated in SH-SY5Y also had a negative fold change in IMR-32, and 6 of the 7 genes that were up-regulated in SH-SY5Y also had a positive fold change in IMR-32. These data show that the expression of the same gene-set was also modified in a second type of neurocyte, although the most sensitive model for detecting the effect of *Gelsemium s.* is the SH-SY5Y cell line.

### Real time quantitative PCR: Validation of the microarray data

To validate the microarray results, RT-qPCR analysis was performed on SH-SY5Y cells exposed to the *Gelsemium s*. 2c and the corresponding control. RT-PCR was carried out on the cDNA obtained from the RNA samples of 3 replicate experiments tested by microarray assay. Among the list of DEGs (see Table 1) we selected 13 genes according to the extent of expression changes and their potential relevant functions (e.g. transcription factors, G-protein coupled receptors or neuropeptides) (Table 2). The genes investigated by quantitative PCR generally confirmed the changes obtained by microarray assay. DDI1, EN2, GALR2, GPR25, OR5C1, Klkbl4 and TAC4 genes were down-regulated in *Gelsemium s*. 2c samples compared to Control 2c in the three replicated experiments. Negative fold changes were observed also with BIRC8 genes, although with variable values. The applied RT-qPCR assays could not detect AIPL1, C1orf167, LST1 and OR4X1, because their expression was under the sensitivity of the assay, and did not confirm the up-regulation of ALK3 gene.

**Table 2:** **Validation of microarray data of selected genes by RT-qPCR in *Gelsemium s*. 2c versus Control 2c treated samples**

		**Fold change microarray**^**1**^					**Fold change RT-PCR**^**2**^				
**Symbol**	**Gene ID**	**R1**	**R2**	**R3**	**Mean**	**SEM**	**R1**	**R2**	**R3**	**Mean**	**SEM**
AIPL1	23746	-0.60	-0.59	-1.06	-0.75	0.13	n.d.	n.d.	n.d.		
ALPK3	57538	1.16	0.04	0.96	0.72	0.28	-0.87	0.24	-0.25	-0.29	0.26
BIRC8	112401	-0.79	-0.77	-0.91	-0.82	0.04	1.64	-1.12	-1.43	-0.30	0.80
C1ORF167	284498	-0.69	-0.51	-1.00	-0.73	0.12	n.d.	n.d.	n.d.		
DDI1	414301	-0.93	0.02	-1.30	-0.74	0.32	-0.62	0.16	-1.23	-0.56	0.33
EN2	2020	-0.62	-0.13	-0.77	-0.51	0.16	-1.53	-0.12	-0.41	-0.69	0.35
GALR2	8811	-0.57	-0.36	-0.72	-0.55	0.08	-0.94	-0.19	-0.61	-0.58	0.18
GPR25	2848	-1.15	0.20	-1.08	-0.68	0.36	-0.74	0.60	-0.11	-0.08	0.32
LST1	221191	-0.71	-0.76	-1.18	-0.88	0.12	n.d.	n.d.	n.d.		
OR4X1	7940	-0.96	-0.55	-0.74	-0.75	0.10	n.d.	n.d.	n.d.		
OR5C1	390113	-0.84	-0.33	-0.79	-0.66	0.13	-0.95	-0.51	-1.00	-0.82	0.13
Klkbl4	392391	-0.05	-0.70	-1.04	-0.60	0.24	-1.10	-0.17	-0.41	-0.56	0.23
TAC4	255061	-0.23	-0.28	-1.34	-0.62	0.30	-1.14	-0.10	-0.46	-0.57	0.25

^1^Fold change was calculated as Log2 ratio between G2c and Ct2c expression values within the three replicated experiments (R1-R3); ^2^fold change was calculated as Log2 transformation of 2-ΔΔCq between G2c and Ct2c. N.d., not detectable.

### Statistical analysis of data from *Gelsemium s*. dilutions and controls

SH-SY5Y cells treated with higher *Gelsemium s.* dilutions (3c, 4c, 5c, 9c, 30c) showed changes in gene expression due to treatment, which were rated by Limma statistics above the 5% of FDR. Inspection of data reported in Table 3, concerning the expression profiles of the 56 DEGs (49 down-regulated and 7 up-regulated by *Gelsemium s.* 2c), highlights small expression changes (i.e. |log2 fold change| from 0.05 to 0.6) in 52, 48, 39, 36 and 48 genes of cells treated with *Gelsemium s*. 3c, 4c, 5c, 9c and 30c respectively. In order to analyze the statistical significance of these effects, a further approach was applied to these 56 DEGs. The hypothesis tested was to determine whether treated samples were different from controls or not and, in particular, if the direction of DEGs’ changes detected in the 2c was maintained across all other dilutions rather than randomly distributed.

**Table 3:** **Fold changes of the 56 differentially expressed genes and the 4 housekeeping transcripts in cells treated with the 6 *Gelsemium s. *** **dilutions compared to means of controls**

**Transcript ID**	**Symbol**	**G 2c**	**G 3c**	**G 4c**	**G 5c**	**G 9c**	**G 30c**
AB032956	GALNTL1	-0.57	-0.20	0.16	-0.13	0.02	0.09
AF000424	LST1	-0.84	-0.18	-0.20	-0.05	-0.14	-0.23
AJ830742	AIPL1	-0.82	-0.43	-0.25	-0.24	-0.07	-0.08
AK058068	Klkbl4	-0.79	-0.41	-0.17	0.11	-0.15	-0.07
AK094115	N/A	-0.70	-0.35	-0.04	-0.01	0.01	-0.41
AK097834	RP5-821D11.2	-0.68	-0.31	-0.03	-0.21	-0.01	0.01
AK128093	N/A	-0.63	-0.28	-0.15	0.05	-0.19	-0.30
AL833920	C1orf167	-0.80	-0.60	0.17	-0.04	0.18	-0.22
AY358368	CDHR5	-0.63	-0.23	-0.09	-0.10	-0.19	-0.25
AY358413	N/A	-0.71	-0.15	0.10	0.04	0.10	-0.28
BC016964	MRGPRF	-0.55	-0.08	-0.12	-0.02	0.06	0.07
BC020658	TMEM40	-0.70	-0.56	-0.15	-0.15	0.04	-0.12
BC039318	BIRC8	-0.76	-0.42	-0.10	-0.09	0.09	-0.02
BC042847	LOC150763	-0.51	-0.29	-0.12	0.01	-0.03	-0.03
BC071746	CLEC7A	-0.54	-0.32	-0.12	-0.17	0.14	0.17
BC101635	NKX6-2	-0.53	-0.56	-0.12	-0.12	-0.14	-0.12
BC104999	CASR	-0.59	-0.17	-0.25	0.08	0.06	-0.30
NM_000066	C8B	-0.53	-0.07	0.00	-0.07	-0.03	-0.23
NM_001001711	DDI1	-0.56	-0.21	-0.27	-0.31	-0.17	-0.26
NM_001001923	OR5C1	-0.71	-0.28	-0.22	0.08	-0.13	-0.24
NM_001003397	TPD52L1	-0.62	-0.31	-0.33	-0.18	0.04	-0.31
NM_001004726	OR4X1	-0.83	-0.34	0.07	0.03	-0.11	-0.18
NM_001427	EN2	-0.51	-0.33	-0.22	-0.06	0.02	-0.13
NM_003857	GALR2	-0.55	-0.31	-0.13	0.02	-0.01	-0.13
NM_004244	CD163	-0.63	-0.30	-0.20	-0.13	0.04	-0.25
NM_005205	COX6A2	-0.64	-0.39	-0.38	-0.07	-0.30	-0.17
NM_005298	GPR25	-0.75	-0.41	0.02	-0.05	-0.02	0.02
NM_006686	ACTL7B	-0.55	-0.44	-0.13	-0.02	-0.01	-0.15
NM_012377	OR7C2	-0.77	-0.22	-0.03	-0.14	-0.14	-0.03
NM_014270	SLC7A9	-0.62	-0.16	-0.20	0.01	-0.27	-0.19
NM_018404	ADAP2	-0.75	-0.40	-0.30	-0.19	0.04	0.03
NM_018965	TREM2	-0.69	-0.34	-0.08	-0.14	0.09	-0.20
NM_138784	RP11-45 J16.2	-0.58	-0.29	0.06	-0.05	0.20	-0.17
NM_145898	CCL23	-0.55	0.03	-0.09	-0.20	0.02	-0.20
NM_170685	TAC4	-0.67	-0.30	-0.19	-0.06	-0.13	-0.24
NM_182510	FLJ32252	-0.52	-0.33	-0.26	-0.10	-0.10	0.02
NM_198690	KRTAP10-9	-0.73	-0.29	-0.10	-0.03	-0.28	-0.13
NM_206895	UNQ830	-0.62	-0.45	-0.22	-0.25	-0.10	-0.18
NM_207478	FLJ44385	-0.68	-0.12	-0.10	-0.08	-0.01	-0.26
X04876	MPO	-0.72	-0.36	-0.20	0.19	-0.01	-0.16
XM_375090	FLJ44817	-0.62	-0.58	-0.16	0.00	-0.27	-0.11
XM_497769	LOC644280	-0.58	-0.19	-0.20	-0.02	-0.07	-0.05
XM_499305	LOC441239	-0.63	-0.31	-0.21	-0.08	-0.15	-0.24
XM_927256	LOC644003	-0.54	-0.57	-0.31	-0.19	-0.27	-0.10
XM_929203	LOC646258	-0.51	-0.20	-0.38	-0.13	-0.11	-0.08
XM_931594	LOC643514	-0.54	-0.23	-0.11	-0.01	-0.21	-0.16
XM_931993	LOC644065	-0.65	-0.29	-0.15	0.05	-0.06	-0.21
XM_934559	LOC647240	-0.60	-0.40	-0.23	-0.16	-0.08	-0.08
XM_935431	LOC374569	-0.54	-0.31	-0.14	-0.12	-0.13	-0.10
AK130941	KIAA0825	0.63	0.30	-0.07	-0.07	-0.06	0.15
NM_001001789	C21orf24	0.52	0.19	0.08	-0.01	0.00	0.21
NM_001024603	LOC154872	0.51	0.13	0.15	0.05	0.06	0.21
NM_001890	CSN1S1	0.57	-0.04	0.03	0.30	-0.07	0.11
NM_018431	DOK5	0.56	0.04	0.03	0.01	-0.02	0.31
NM_020482	FHL5	0.55	0.38	0.01	0.10	-0.02	-0.08
NM_020778	ALPK3	0.76	0.45	0.23	0.16	0.03	0.19
BC001601	GAPDH^1^	0.01	0.09	0.02	0.10	0.03	0.04
NM_002046	GAPDH^1^	0.09	-0.14	-0.02	-0.10	-0.01	0.00
BC009081	GAPDH^1^	0.01	-0.05	0.01	-0.04	-0.04	-0.03
NM_001101	ACTB^1^	-0.04	-0.05	-0.05	0.05	0.02	0.00

N = 4 experiments. ^1^Housekeeping transcripts.

#### Statistical inference

**Figure 5:**
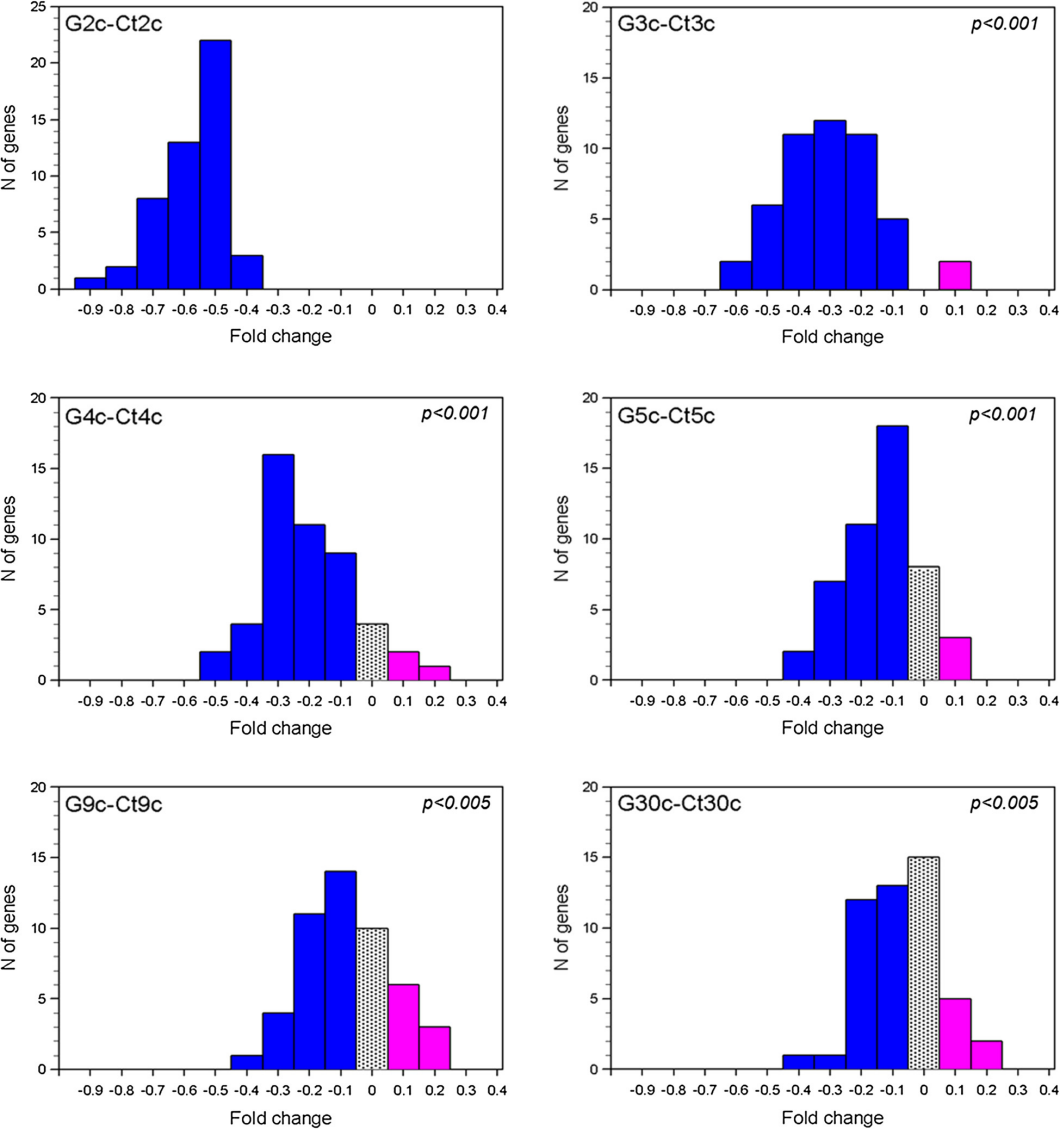
**Frequency of fold change values in the down-regulated gene-set after *Gelsemium s. *treatments.** In this analysis the 49 genes whose expression was down-regulated by *Gelsemium s.* 2c were considered. Mean Log2 fluorescence values from *Gelsemium s.*-treated samples (Gnc) and those from controls (Ctnc) were obtained from 4 microarray experiments and their difference was considered as fold change attributable to *Gelsemium s.* effect (see Methods). Absolute fold changes less than or equal to 0.05 were considered null. Blue bars: frequencies of genes with negative fold change (< -0.05); grey bars: frequency of unaffected genes (from -0.05 to 0.05); pink bars: frequencies of genes with positive fold change (> 0.05). Fisher exact *p* values are reported in each panel except the G2c-Ct2c that are significant by definition.

**Figure 6:**
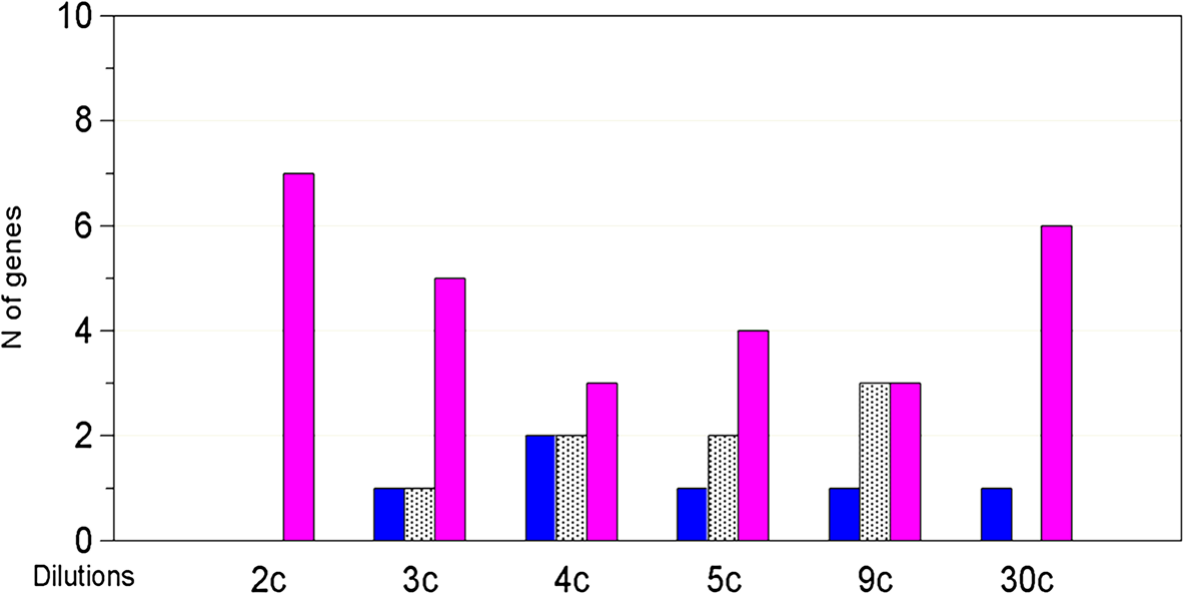
**Number of genes modulated by *Gelsemium s. *dilutions in the panel of up-regulated genes.** In this analysis the 7 genes whose expression was up-regulated by *Gelsemium s.* 2c were considered. Differences less than or equal to 0.05 were considered null. Blue bars: number of genes with negative fold change (< -0.05); grey bars: number of unaffected genes (from -0.05 to 0.05); pink bars: number of genes with positive fold change (> 0.05). Fisher exact test is not significant in any dilution except in the 2c dilution that is significant by definition.

Expression values (mean of 4 experiments) of the 49 down- and 7up-regulated genes referred to the same dilution (2c ÷ 30c) of treatments and respective controls were compared. Additional file 1 reports Log2 data of all samples tested in this analysis. Friedman test estimated the overall variance among the samples and showed that the value distributions of the 12 different treatment groups (6 *Gelsemium s.* and 6 controls, n = 49 or n = 7 data for down-regulated and up-regulated genes, respectively) are significantly different (*p < 0.0001*). For a direct evaluation of the differences between *Gelsemium s.* treatments and the corresponding controls, Figure 5 shows the distribution of the fold changes in the 49 down-regulated genes for all the dilutions tested. Even though the size of the differences was distributed in a small range, the number of genes with negative fold change (Log2 *Gelsemium s.* < Log2 control, blue in Figure 5) was systematically higher than the number of genes with positive fold change (Log2 *Gelsemium s.* > Log2 control, pink bars in Figure 5). In particular, the frequency of down-regulated vs up-regulated genes was 49 vs. 0 (100% vs. 0%) in 2c, as expected, 47 vs. 2 (96% vs. 4%) in 3c, 42 vs. 3 (86% vs. 6%) in 4c, 38 vs. 3 (78% vs. 6%) in 5c, 30 vs. 9 (61% vs. 18%) in 9c, 27 vs 7 (55% vs. 14%) in 30c. By applying Fisher exact test, the exact probability of the distributions, under the null hypothesis of indifference, was calculated and significant *p* values resulted for all dilutions (*p < 0.001* for 3c, 4c and 5c treatments, *p = 0.0035* for 9c and *p = 0.004* for 30c). The absence of an equal scattering between the two signs (positive and negative fold changes) suggests that *Gelsemium s.* at high dilutions affects the expression of a significant portion of these genes. This conclusion is reinforced by a separate Fisher exact test carried out on a list of 49 genes randomly selected by the SPSS software from the 45033 transcripts (excluding the 56 DEGs); as reported in Additional file 2, no significantly different distribution of down-regulated or up-regulated genes in this random gene-set was observed with any *Gelsemium s.* dilution. Figure 6 reports the results for the panel of 7 up-regulated genes. Due to the small number of these genes, a distribution of fold changes could not be drawn and the statistical power of analysis was low. By Fisher exact test, a statistically significant prevalence of positive fold changes was observed only in 2c, as expected, while the prevalence of positive fold changes in the other dilutions was not significant.

#### Cluster analysis

**Figure 7:**
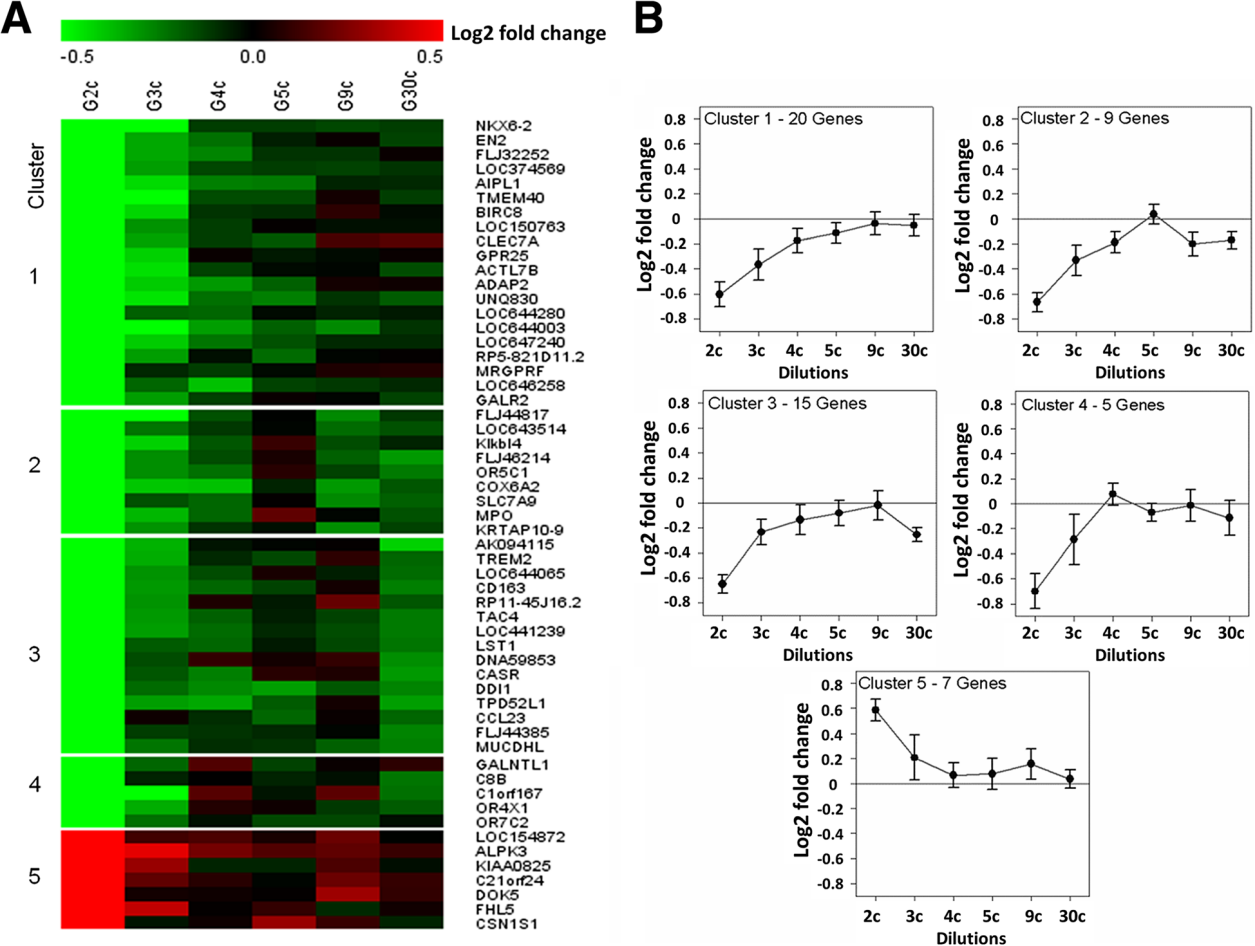
**K-mean clustering of the genes modulated upon exposure to *Gelsemium s. *dilutions.** The expression profile of 56 genes significantly modulated by *Gelsemium s.* 2c was evaluated also upon exposure to increasing *Gelsemium s.* (G) dilutions. Fold change was calculated as the difference between Log2 fluorescence values of each *Gelsemium s.* dilution and the mean Log2 fluorescence of the controls. Data are means of 4 replicate experiments. **A**. K-mean clusters (KMC) visualized as a colour-coded heat map. The down-regulated genes (green) with similar expression profiles were grouped in 4 clusters and the up-regulated genes (red) in one cluster. **B**. Centroid graphs of the mean fold change of genes in the 5 clusters obtained in KMC analysis.

With the aim to describe the trends of gene expression when exposed to higher *Gelsemium s.* dilutions, k-means cluster analysis was applied on the Log2-fold change profiles of the 56 selected genes. The effect of all the tested *Gelsemium s.* dilutions was visualized as a heat map (Figure 7A) and as mean fold changes in each cluster of genes (Figure 7B). This allowed to identify gene subsets with similar expression profiles, and to detect some trends in the changes induced by increasing *Gelsemium s.* dilutions. Most of the genes down-regulated in the 2c-treated samples were also under-expressed in 3c and, to a varying extent, even in higher dilutions. The frequency of genes with negative fold changes was above 65% in all conditions, and in the sample treated with *Gelsemium s.* 30c the number of common genes that were down-regulated in all dilutions was 20 out of 49 (41%). Cluster 1 contains 20 genes whose expression was down-regulated by the 2c dilution but which were less sensitive to higher dilutions, thus drawing a curve with asymptotic direction. Clusters 2 and 3 group together the genes also down-regulated by the *Gelsemium s.* high dilutions (but on which 5c or 9c, respectively, had no effect), while cluster 4 includes the genes that were clearly responsive to *Gelsemium s.* 2c and 3c only. Cluster 5 contains the 7 up-regulated genes. Though significant up-regulation occurred only with 2c, most of those genes showed a similar effect trend in all dilutions.

### Functions of the modulated genes

To obtain a functional classification, the 56 genes whose expression changed following exposure to *Gelsemium* in SH-SY5Y cells were subjected to analysis of the enriched annotation terms associated with the list. Table 4 reports the top enriched biological themes, particularly the GO terms discovered in the gene list by the DAVID software. A total of 28 genes (all down-regulated) from the list were classified into functional-related gene groups, while 17 IDs were unmapped in the DAVID database (see Table 1) because they have unknown functions. The remaining genes (3 up-regulated and 8 down-regulated) have known functions but were not rated as enriched in the list compared to the whole human transcriptome. The main group of functional features includes genes coding for membrane receptors, and in particular involved in G-protein coupled receptor (GPCR) transduction systems (OR4X1, CASR, OR5C1, CCL23, GPR25, GALR2, OR7C2, MRGPRF). Among these receptors, three have specific functions in olfactory transduction, attuned to detecting different types of stimuli including molecular vibrations [47]. The other clusters of genes may have a role in calcium signaling, inflammatory pathways, neuropeptide/receptor systems or as transcription factors. Of particular relevance for neuronal functions is the small but significant down-regulation of the gene TAC4 and GALR2. The first gene codes for the neuropeptide hemokinin-1 an analogous of substance-P [48], and the second for the receptor 2 of the neuropeptide galanin. Both are involved in the complex system of psyco-neuro-immune-endocrine axis which correlates the emotional responses with the hormone release and the immune functions [49,50].

**Table 4:** **Top enriched annotation terms associated with the 56 genes differentially expressed upon exposure to *Gelsemium s. *** **2c in SH-SY5Y cells**

**Category**	**Annotation term**	**P value**	**Genes**	**Fold enrichment**
SP_PIR_KEYW	receptor	8.32E-11	AIPL1, OR4X1, CASR, OR5C1, GPR25, GALR2, OR7C2, MRGPRF, CLEC7A, TREM2	3.68
GOTERM_BP_FAT	GO:0007186 ~ G-protein coupled receptor protein signalling pathway	6.60E-11	OR4X1, CASR, OR5C1, CCL23, GPR25, GALR2, OR7C2, MRGPRF, TAC4	4.17
GOTERM_BP_FAT	GO:0007166 ~ cell surface receptor linked signal transduction	0.001	OR4X1, CASR, OR5C1, CCL23, DOK5, GPR25, GALR2, OR7C2, MRGPRF, CLEC7A, TAC4	0.08
GOTERM_BP_FAT	GO:0051606 ~ detection of stimulus	0.02	AIPL1, CASR, CLEC7A	13.23
INTERPRO	IPR017452:GPCR, rhodopsin-like superfamily	0.009	OR4X1, OR5C1, GPR25, GALR2, OR7C2, MRGPRF	4.44
KEGG_PATHWAY	hsa04740:Olfactory transduction	0.09	OR4X1, OR5C1, OR7C2	5.31
GOTERM_BP_FAT	GO:0006954 ~ inflammatory response	0.02	C8B, CCL23, CLEC7A, CD163	6.40
GOTERM_BP_FAT	GO:0006952 ~ defense response	0.02	C8B, CCL23, MPO, CLEC7A, CD163	4.23
GOTERM_BP_FAT	GO:0006955 ~ immune response	0.03	C8B, CCL23, LST1, CLEC7A, TREM2	3.77
GOTERM_BP_FAT	GO:0006874 ~ cellular calcium ion homeostasis	0.04	CASR, CCL23, GALR2	8.53
GOTERM_BP_FAT	GO:0030182 ~ neuron differentiation	0.04	LST1, NKX6-2, GALR2, EN2	4.75
GOTERM_CC_FAT	GO:0005886 ~ plasma membrane	0.02	CASR, OR5C1, SLC7A9, MRGPRF, CDHR5, CD163, C8B, OR4X1, ADAP2, GALR2, GPR25, OR7C2, CLEC7A, TREM2	1.65
GOTERM_CC_FAT	GO:0005576 ~ extracellular region	0.071	KLKBL4, C8B, CCL23, MPO, TAC4, TREM2, CSN1S1, CD163	2.03
SP_PIR_ KEYW	Disulfide bond	1.06E-12	KLKBL4, OR5C1, ALPK3, SLC7A9, GALNTL1, CSN1S1, CD163, C8B, OR4X1, CCL23, GALR2, OR7C2, MPO, CLEC7A, TREM2	2.99
SP_PIR_ KEYW	Glycoprotein	0.06	KLKBL4, CASR, OR5C1, MRGPRF, CDHR5, CD163, C8B, OR4X1, GALR2, OR7C2, MPO, CLEC7A, TREM2	1.75

The analysis was performed by DAVID Bioinformatics Resources 6.7 (http://david.abcc.ncifcrf.gov). The genes associated with annotation terms with enrichment *p < 0.1* are reported here.

## Discussion

Natural remedies are increasingly viewed as potentially valuable complements to conventional drugs in integrated treatment strategies for a number of disorders, and many consumers use natural health products alongside prescription medications [51]. Anxiety and depression are among the ailments most frequently reported by patients seeking complementary and alternative medical remedies and/or naturopathic care [9,52,53]. *Gelsemium s.* is a traditional remedy used in complementary and alternative therapies for treating patients who exhibit neurological complaints such as headache and anxiety-like symptoms [9,52,53], but evidence-based clinical studies are few and with contrasting results [53,54].

Homeopathy is a 200-year-old therapeutic system that uses extremely small doses of various substances to stimulate auto-regulation and self-healing processes [55]. Although some conventional physicians find such notions implausible [56], use of highly diluted drugs from homeopathic pharmacopoeia has recently seen a worldwide revival [57,58] and laboratory investigations are increasing in this field [26,27], but scientific evidence of underlying molecular mechanisms is still lacking. Moreover, the experimental approaches adopted to study these remedies, particularly for highly diluted solutions, have suffered from problems with replicability between different laboratories. It is therefore important for any results in this field to be confirmed and consolidated through further investigations by independent laboratories, using rigorous protocols and statistical evaluations. The expression microarray analysis on whole genome, as other high-throughput technologies assisted by bioinformatics, could provide a strong clue as to the mechanism of action and the biological relevance of ultra-low doses and high dilutions interactions.

This is the first comparative transcriptomics approach to investigate changes in the human neurocytes induced by a natural plant remedy, traditionally used for anxiolytic-like effects. The chief innovation of our experimental design is that it employs a wide range of doses/dilutions. This enabled us to explore changes in gene expression from low dilutions (2c or 3c), where the active substances can still be expected to exert their normal pharmaceutical action, to high dilutions (9c or 30c), where the most controversial principles of high dilution pharmacology come into play. Thus, both conventional and 'alternative’ pharmacological theories were evaluated and compared in the same investigation. In previous recent trials, *Gelsemium s.* showed anxiolytic-like effects in mouse emotional response models and appeared to work even at the high dilutions 9c and 30c [26,27]. Two other studies have also found that high dilutions of *Gelsemium s.* exert a preventive action against experimental stress (electric shock) in mice [29] and against convulsions provoked by lithium and pilocarpine in rats [5]. Other researchers have reported an anti-anxiety activity of *Gelsemium s.*[12] and of the alkaloids gelsemine, koumine, and gelsevirine [14,16], but have not explored the effect of ultra-low doses and high dilutions/dynamizations.

To follow up on these in vivo studies, we decided to investigate the action of *Gelsemium s.* at the cellular and transcriptional level. We adopted a validated microarray protocol and applied it to a series of replicate experiments designed to test: a) the null hypothesis that the effect of any *Gelsemium s.* dilutions is similar to that of the control vehicle, b) whether any dose-dependence of the putative effects can be demonstrated. As our model system, we chose the SH-SY5Y and IMR-32 neurocyte cell lines because these have been previously employed for investigations of natural compounds [31], neurotrophic factors [32], mood stabilisers [59], and antipsychotics [33]. In our conditions, this line proved to be more responsive to *Gelsemium s.* than IMR-32 and was used to compare the effects of high dilutions. The cells were functional, as demonstrated by intracellular calcium increase following treatment with the neurotransmitter carbachol, and none of the *Gelsemium s.* dilutions affected their growth rate or metabolic activity.

### Low-dilution effects

The most evident and statistically significant modification of cellular gene expression was induced by the lowest dilution of the medicine that we tested, namely *Gelsemium s.* 2c, as is to be expected with a dose-dependent effect. Spectroscopic analysis of the tested samples confirmed that the starting 1c solution supplied by manufacturer contained a considerable amount of original extract compounds, and proportionate quantities were also detected in the 2c dilutions prepared both by the manufacturer and in our laboratory. However, dilution equal or beyond 3c brought the concentration of detectable compounds below the minimum sensitivity threshold of optical spectroscopy.

Although *Gelsemium s.* contains several different compounds [2,3], the major active alkaloid of this plant is gelsemine, which was present in a concentration of 6.5 × 10^-9^ M in the final incubation mixture of cells treated with *Gelsemium s.* 2c. This nanomolar dosage is much lower than the toxic doses that have been reported in poisoning cases [60] and in experimental evaluations of LD50 [14]. In fact, *Gelsemium s.* 2c at the lowest dilution (highest dose) tested in this model system did not cause cell toxicity or viability impairment. This evidence is in agreement with recent hypotheses explaining the homeopathic effects (in the range of very low doses) in the framework of hormesis, where substance which are toxic at high doses turn into therapeutic when diluted to low and ultra-low doses [61]. According to the hormetic theory, ultra-diluted drugs and nanoparticles will act as low-dose stress conditions that could possibly evoke an adaptive response process producing effects that might modulate gene expression [62-64].

The effects on gene expression observed here are specifically targeted to the regulation of certain functions, possibly linked with the plant’s pharmacological activity. Of a total of 45033 transcripts, 49 were down-regulated and 7 up-regulated by the 2c dilution. This effect was quantitatively small (absolute value of fold change between 0.5 and 1.0) but statistically significant (adjusted *p < 0.05*). In general, the prevalence of down-regulation seems to indicate a tendency to reduce cell excitability, especially because several of the genes in question belong to surface receptors involved in GPCR signaling and calcium homeostasis. Moreover, this first microarray screening of the effects of *Gelsemium s.* on neurocytes revealed a significant down-regulation of genes for inflammatory response, olfactory transduction and neuron differentiation. Clearly, this plant species contains a variety of active chemical principles which are presumably involved in different pathways of cell regulation besides the pure neural function, as suggested by reports of possible anticancer and immunomodulating activities [17-19].

A hypothetical neurological target of *Gelsemium s.* has been suggested by studies showing that gelsemine stimulates the biosynthesis of allopregnanolone in the rat brain [30,65], but the genes of neurosteroid enzymatic pathways were not modulated in our cell system. This apparent discrepancy may depend either on the fact that we used a cell line, whereas Venard et. al. [30,65] used slices of spinal cord and limbic system, or on the fact that they studied a post-translational level of regulation, linked to enzyme function and not to gene expression. In any case, since in our model the effects of *Gelsemium s.* were quantitatively small, as confirmed by RT-PCR results, no definite conclusions regarding the role of single genes in the action mechanism of this plant can be drawn at this stage.

These microarray findings can be regarded as a preliminary screening of the sensitivity of SH-SY5Y cellular system to *Gelsemium s.*, while more robust conclusions about the possible role of the implicated genes will require to determine whether proteins encoded by the affected genes are similarly changed, through proteomic and phosphoproteomic approaches, and/or further studies using plant purified active compounds.

### Ultra-low doses and high dilutions

The second major goal of this investigation was to study the dose-effect relationship, which is of central importance in any kind of pharmacological approach. As noted above, the *Gelsemium s.* 2c dilution yielded statistically significant results for 56 genes. This raised the question of whether those same genes, which appeared to be most sensitive to *Gelsemium s.,* would also be modified by higher dilutions. Since the quantitative changes for the 3c and higher dilutions were quite low (Table 3 and Figure 7B), the 4 replicates were insufficient to yield statistical confidence for analysis of single transcripts. We therefore employed cluster analysis to separately describe the trends of 6 gene subsets with similar expression profiles. All 4 down-regulated clusters included genes with negative mean fold changes, though of varying magnitude. Most notably, we found two clusters (2 and 3 in Figure 7) that included a total of 24 genes clearly responsive to *Gelsemium s.* 30c, and characterized by a bell-shaped dilution-effect curve. Exploring results accurately (Table 3), some genes showed an interesting pattern of expression in function of *Gelsemium* dilution. For instance, the EN2 gene that was under-expressed in treated cells exhibited a bell-shaped curve. This tendency can be seen in other genes in the cluster analysis. Moreover, it seems that after 9c, another wave of expressions or no-expressions is recovered. Maybe future testing even higher dilutions, such as 100c or 200c, the bell-shaped curve could be more evident and, thus, the hypothesis of ultra-sensible genes could be checked.

For the high dilutions, due to the small changes of gene expression, the only hypothesis statistically evaluable is the global effect of *Gelsemium s.* dilutions on the 49 down-regulated and 7 up-regulated genes, considered as gene-sets. Using the Fisher exact test (*Gelsemium s.* dilutions vs. their respective control solutions), the null hypothesis was rejected for every dilution in the down-regulated gene-set. This outcome of our microarray analysis is astounding if we consider that the 9c and 30c dilutions were obtained from MT extract by dilution factors of 10^18^ and 10^60^ respectively. Starting from a crude MT containing the active principle gelsemine at a concentration of 6.5 × 10^-4^ M, the 9c dilution would theoretically contain 6.5 × 10^-22^ M gelsemine, corresponding to less than 1 molecule per ml in the final working solution; even in the case of the 5c dilution, where the theoretical gelsemine concentration is 6.5 × 10^-15^ M, it can be calculated that this would correspond to 3.9 × 10^7^ molecules per culture plate, i.e. about 13 molecules per seeded cell. These results suggest that neurocytes have a number of genes with extreme sensitivity to *Gelsemium s.* effects, even if those effects of high dilutions are quantitatively very small (decrease in expression by approximately 10% to 20% compared to the control). The physiological or pharmacological implications of this observation remain to be clarified, but the rejection of the null hypothesis furnishes a new input for the open debate on this kind of therapeutic approach.

### Technical issues and confounding factors

The puzzling evidence of gene expression changes under the influence of homeopathic dilutions prompt an analysis of the possible confounding factors that might explain the effects observed. We adopted different measures to address the issue of possible experimental artifacts. To avoid dye-bias artifacts a single-channel microarray was employed. We adopted a microarray design with probes of the same probe-set located in not contiguous positions on the array, so that artifacts due to uneven hybridization would only affect a subset of probes for a probe-set. Anyhow, the absence of spatial biases in fluorescence signal was assessed by checking the coefficient of variation of the mean signal intensities of different portions of each array. The experimental set up could have introduced biases and “position effects” if handling of control and *Gelsemium s.* matched dilutions was not equivalent. Actually, we conducted four independent experiments in which *Gelsemium s.* dilutions and the corresponding vehicle controls were processed in tandem (from drug addition to RNA extraction and cDNA synthesis). In every subarray of the chip, each transcript was targeted with three separate probes, merging the fluorescence values and attributing a statistical score.

Regarding the statistical analysis, the large number of genes of the complete set causes some problems concerning the choice of “interesting” genes. The approach followed here was quite stringent and limited the number of genes considered, reducing the probability of “false positive” results, but forcing to discard some possibly interesting genes from the analysis. Moreover, the small entity of the expression changes observed with high dilutions unavoidably reduced statistical inference in the single genes, especially since multiplicity corrections were applied. The choice of analyzing the sign of the fold changes in a pool of genes, rather than the variance of a single gene, may lead to a loss of statistical information, to the advantage of greater precision in discarding the null hypothesis. Further research specifically oriented on the most responsive genes, with suitable sample sizes, could possibly overcome this limitation of the microarray approach.

### Physico-chemical and biological hypotheses

Our results are in keeping with a number of experimental observations from a variety of research fields, confirming that highly diluted compounds exert statistically significant effects on biological systems [66-69]. Thus far there is no satisfactory or unifying theoretical explanation for these claims, though some have hypothesized that the dynamics of the solvent water (or water-ethanol) on a mesoscopic scale may play a part [70]. Three major models for how this happens are currently being investigated: the water clusters or clathrates, the coherent domains postulated by quantum electrodynamics, and the formation of nanoparticles from the original solute plus solvent components. It has been suggested that a major role in the formation of water clusters is played by silica released from the glass containers which are usually employed in the preparation of homeopathic drugs [71]. Silica nanostructures formed during succussion in glass and/or biosynthesized by specific plant extract tinctures may also acquire and convey epitaxial information from the remedy source materials into the higher potencies [21,72,73]. In our experimental model, since the *verum* were succussed samples, we used the succussed ethanol/water solutions as negative controls and evaluated preliminarily the variability of the negative system before assessing the biological effect of the succussed/diluted drug. Notably, in our experiments serial dilutions/succussions were performed in glass bottles, with the exception of the last step, which was developed in polystyrene tubes. Thus the hypothetical role of silicates in nanoparticle formation is pertinent, but also the contribution of polystyrene should not be excluded [74].

Recent evidence supports the plausibility that homeopathic *Gelsemium s*. in the potencies tested could contain crudely formed nanoparticles. Bel-Haaj et al. [75] demonstrated that just extended ultrasonication of plant starch can create starch nanoparticles in water. Moreover, electron microscopic evidence of nanoparticles has been obtained in several different plants prepared homeopathically [76]. *Gelsemium* mother tincture itself, like many other plant extracts, can biosynthesize nanoparticles of silver metal from precursor substrate [77]. Nanoparticles have unique biological and physicochemical properties, including increased catalytic reactivity, protein and DNA adsorption, bioavailability, dose-sparing, electromagnetic, and quantum effects that are different from those of bulk-form materials [23]. As an example, Prakash and colleagues [78] compared in model animals the anti-anxiety effects of hypericum prepared as gold nanoparticles versus a bulk form and observed more significant effects with the nano-hypericum, even at a 10-fold lower dose. Higher cellular uptake of nano-encapsulated (poly lactide-co-glycolide) *Gelsemium s.* than of its bulk form has been observed by Bhattacharyya et al. [79].

The hypotheses regarding the possible biological mechanisms of highly diluted/dynamized solutions (beyond Avogadro-Loschmidt limit) at the level of DNA expression variously invoke sensitivity to bioelectromagnetic information, participation of water chains in signaling, stochastic resonance, and regulation of bifurcation points of nonlinear systemic networks [64,80-83]. Based on microarray data, it has been suggested that gene regulatory networks may be regarded as dynamically 'critical’ systems poised near the phase transition between order and chaos [80,84,85], where extreme sensitivity to initial conditions and small perturbations is well known to occur. Chaotic regimes have been found in a number of physiological systems, including neural systems [86-88], and this would result in enhanced susceptibility to extremely low energy inputs and to small changes of regulatory factors. According to this argument, the highly diluted drug might be regarded as a solution endowed with water clusters and/or nanoparticulate structures capable of communicating some pharmacological information, through a resonance process, to biological fluids and to cell critical systems such as macromolecules, alpha-helixes, filamentous structures, receptors and DNA networks. This effect could be mediated by the participation of a dynamic intracellular water network which may be presumed to exist in living cells [89].

## Conclusions

This study provides evidence that *Gelsemium s.* exerts a prevalently inhibitory effect on a series of neurocyte genes across a wide dose-range. The effect decreases with increasing dilutions, but whole genome expression analysis allowed to detect statistically significant changes even at the highest dilutions tested (9c and 30c). The results suggest the extreme sensitivity of human gene expression to regulation by ultra-low doses and high dilutions/dynamizations of a plant remedy and encourage further efforts in research on this field. Studies using “omic-based” approaches and systems biology should be particularly worthy at generating new hypotheses on mechanisms for the effects of highly diluted natural compounds.

## Competing interests

The authors declare that they have no competing interests.

## Authors’ contributions

PB conceived the experiments, MM, DO and MC designed and performed the experiments, PT, MB, MM, and DO analyzed the data, MM and PB wrote the paper. All authors read and approved the final manuscript.

## Pre-publication history

The pre-publication history for this paper can be accessed here:

http://www.biomedcentral.com/1472-6882/14/104/prepub

## Supplementary Material

Additional file 1 **Microarray expression values of 56 transcripts in SH-SY5Y neurocytes treated with *Gelsemium s. *or control dilutions.** Data are reported as Log2 transformed fluorescence values from four replicate microarray assays. Click here for file

Additional file 2 **Frequency of fold change values in a randomly chosen gene-set after *Gelsemium s. *treatments.** A list of 49 genes was generated by randomized selection from the whole transcriptome using SPSS software (excluding the differentially expressed genes) and fold change was calculated from the difference of mean Log2 fluorescence values of *Gelsemium s.*-treated samples (Gnc) vs those of controls (Ctnc). Absolute fold changes less than or equal to 0.05 were considered null. Blue bars: frequencies of genes with negative fold change (< -0.05); grey bars: frequency of unaffected genes (from -0.05 to 0.05); pink bars: frequencies of genes with positive fold change (> 0.05). For these randomly selected genes, Fisher exact test is not significant in any dilution. Click here for file

## References

[B1] Schun Y Cordell GA Cytotoxic steroids of Gelsemium sempervirens J Nat Prod 1987 50 195 198 10.1021/np50050a012 3655795

[B2] Dutt V Thakur S Dhar VJ Sharma A The genus Gelsemium: an update Pharmacogn Rev 2010 4 185 194 10.4103/0973-7847.70916 22228960 PMC3249920

[B3] Jin GL Su YP Liu M Xu Y Yang J Liao KJ Yu CX Medicinal plants of the genus Gelsemium (Gelsemiaceae, Gentianales)-a review of their phytochemistry, pharmacology, toxicology and traditional use J Ethnopharmacol 2014 doi:10.1016/j.jep.2014.01.003 10.1016/j.jep.2014.01.003 24434844

[B4] Valnet J Phytothérapie 1992 Paris: Maloine

[B5] Peredery O Persinger MA Herbal treatment following post-seizure induction in rat by lithium pilocarpine: Scutellaria lateriflora (Skullcap), Gelsemium sempervirens (Gelsemium) and Datura stramonium (Jimson Weed) may prevent development of spontaneous seizures Phytother Res 2004 18 700 705 10.1002/ptr.1511 15478209

[B6] Boericke W Materia Medica with Repertory 1927 Philadelphia: Boericke & Tafel, Inc

[B7] Binsard AM Guillemain J Platel A Savini EC Tetau M Etude psycho-pharmacologique de dilutions homéopathiques de Gelsemium et d’Ignatia Ann Homeop Fr 1980 22 35 50

[B8] Guillemain J Rousseau A Dorfman P Tetau M Recherche en psychopharmacologie Cah Biother 1989 103 53 66

[B9] Guermonprez M Homéopathie, Principles - Clinique - Techniques 2006 Paris: CEDH

[B10] Bellavite P Magnani P Marzotto M Conforti A Assays of homeopathic remedies in rodent behavioural and psychopathological models Homeopathy 2009 98 208 227 10.1016/j.homp.2009.09.005 19945676

[B11] Gahlot K Abid M Sharma A Pharmacological evaluation of *Gelsemium sempervirens* roots for CNS depressant activity Int J Pharm Tech Res 2012 3 693 697

[B12] Dutt V Dhar VJ Sharma A Antianxiety activity of Gelsemium sempervirens Pharm Biol 2010 48 1091 1096 10.3109/13880200903490521 20860436

[B13] Liu M Shen J Liu H Xu Y Su YP Yang J Yu CX Gelsenicine from Gelsemium elegans attenuates neuropathic and inflammatory pain in mice Biol Pharm Bull 2011 34 1877 1880 10.1248/bpb.34.1877 22130245

[B14] Liu M Huang HH Yang J Su YP Lin HW Lin LQ Liao WJ Yu CX The active alkaloids of Gelsemium elegans Benth. are potent anxiolytics Psychopharmacology (Berl) 2013 225 839 851 10.1007/s00213-012-2867-x 23052566

[B15] Zhang JY Gong N Huang JL Guo LC Wang YX Gelsemine, a principal alkaloid from Gelsemium sempervirens Ait., exhibits potent and specific antinociception in chronic pain by acting at spinal alpha3 glycine receptors Pain 2013 154 2452 2462 10.1016/j.pain.2013.07.027 23886522

[B16] Meyer L Boujedaini N Patte-Mensah C Mensah-Nyagan AG Pharmacological effect of gelsemine on anxiety-like behavior in rat Behav Brain Res 2013 253 90 94 23850351 10.1016/j.bbr.2013.07.010

[B17] Bhattacharyya SS Mandal SK Biswas R Paul S Pathak S Boujedaini N Belon P Khuda-Bukhsh AR In vitro studies demonstrate anticancer activity of an alkaloid of the plant Gelsemium sempervirens Exp Biol Med (Maywood) 2008 233 1591 1601 10.3181/0805-RM-181 18997108

[B18] Zhao QC Hua W Zhang L Guo T Zhao MH Yan M Shi GB Wu LJ Antitumor activity of two gelsemine metabolites in rat liver microsomes J Asian Nat Prod Res 2010 12 731 739 10.1080/10286020.2010.492951 20839118

[B19] Rammal H Soulimani R Effects of high doses of Gelsemium sempervirens L. on GABA receptor and on the cellular and humoral immunity in mice J Med Med Sci 2010 1 40 44

[B20] Xu YK Liao SG Na Z Hu HB Li Y Luo HR Gelsemium alkaloids, immunosuppressive agents from Gelsemium elegans Fitoterapia 2012 83 1120 1124 10.1016/j.fitote.2012.04.023 22579843

[B21] Roy R Tiller W Bell IR Hoover MR The structure of liquid water. Novel insights from materials research; potential relevance to homeopathy Mat Res Innovat 2005 9 98 103

[B22] Yinnon TA Yinnon CA Electric dipole aggregates in very diluted polar liquids: theory and experimental evidence Int J Mod Phys B 2011 25 3707 3743 10.1142/S0217979211101624

[B23] Bell IR Koithan M A model for homeopathic remedy effects: low dose nanoparticles, allostatic cross-adaptation, and time-dependent sensitization in a complex adaptive system BMC Complement Altern Med 2012 12 191 10.1186/1472-6882-12-191 23088629 PMC3570304

[B24] Endler P Thieves K Reich C Matthiessen P Bonamin L Scherr C Baumgartner S Repetitions of fundamental research models for homeopathically prepared dilutions beyond 10(-23): a bibliometric study Homeopathy 2010 99 25 36 10.1016/j.homp.2009.11.008 20129174

[B25] Stock-Schroer B Albrecht H Betti L Dobos G Endler C Linde K Lüdtke R Musial F van Wijk R Witt C Baumgartner S Reporting experiments in homeopathic basic research-description of the checklist development Evid Based Complement Alternat Med 2011 2011 639260 19884113 10.1093/ecam/nep170 PMC3136753

[B26] Magnani P Conforti A Zanolin E Marzotto M Bellavite P Dose-effect study of Gelsemium sempervirens in high dilutions on anxiety-related responses in mice Psychopharmacology (Berl) 2010 210 533 545 10.1007/s00213-010-1855-2 20401745 PMC2877813

[B27] Bellavite P Conforti A Marzotto M Magnani P Cristofoletti M Olioso D Zanolin ME Testing homeopathy in mouse emotional response models: pooled data analysis of two series of studies Evid Based Complement Alternat Med 2012 2012 954374 22548123 10.1155/2012/954374 PMC3324905

[B28] Sukul NC Bala SK Bhattacharyya B Prolonged cataleptogenic effects of potentized homoeopathic drugs Psychopharmacology (Berl) 1986 89 338 339 3088660 10.1007/BF00174371

[B29] Bousta D Soulimani R Jarmouni I Belon P Falla J Foment N Younos C Neurotropic, immunological and gastric effects of low doses of Atropa belladonna L., Gelsemium sempervirens L. and Poumon histamine in stressed mice J Ethnopharmacol 2001 74 205 215 10.1016/S0378-8741(00)00346-9 11274819

[B30] Venard C Boujedaini N Mensah-Nyagan AG Patte-Mensah C Comparative analysis of gelsemine and Gelsemium sempervirens activity on neurosteroid allopregnanolone formation in the spinal cord and limbic system Evid Based Complement Alternat Med 2011 407617 doi:10.1093/ecam/nep083 19628662 10.1093/ecam/nep083 PMC3136435

[B31] Seo JJ Lee SH Lee YS Kwon BM Ma Y Hwang BY Hong JT Oh KW Anxiolytic-like effects of obovatol isolated from Magnolia obovata: involvement of GABA/benzodiazepine receptors complex Prog Neuropsychopharmacol Biol Psychiatry 2007 31 1363 1369 10.1016/j.pnpbp.2007.05.009 17698274

[B32] Donnici L Tiraboschi E Tardito D Musazzi L Racagni G Popoli M Time-dependent biphasic modulation of human BDNF by antidepressants in neuroblastoma cells BMC Neurosci 2008 9 61 10.1186/1471-2202-9-61 18601743 PMC2483719

[B33] Park SW Seo MK Cho HY Lee JG Lee BJ Seol W Kim YH Differential effects of amisulpride and haloperidol on dopamine D2 receptor-mediated signaling in SH-SY5Y cells Neuropharmacology 2011 61 761 769 10.1016/j.neuropharm.2011.05.022 21663752

[B34] Fisher P What is homeopathy? An introduction Front Biosci (Elite Ed) 2012 4 1669 1682 22201984 10.2741/e489

[B35] Anonymous Pharmacopée Homéopathique Française - X édition 2002 Saint-Denis Cedex (FR): Agence Française de Sécurité Sanitaire de Produits de Santé

[B36] Kohl RL Perez-Polo JR Quay WB Effect of methionine, glycine and serine on serine hydroxymethyltransferase activity in rat glioma and human neuroblastoma cells J Neurosci Res 1980 5 271 280 10.1002/jnr.490050403 6776288

[B37] Chakravarthy B Gaudet C Menard M Atkinson T Brown L Laferla FM Armato U Whitfield J Amyloid-beta peptides stimulate the expression of the p75(NTR) neurotrophin receptor in SHSY5Y human neuroblastoma cells and AD transgenic mice J Alzheimers Dis 2010 19 915 925 20157247 10.3233/JAD-2010-1288

[B38] Ishiyama M Tominaga H Shiga M Sasamoto K Ohkura Y Ueno K A combined assay of cell viability and in vitro cytotoxicity with a highly water-soluble tetrazolium salt, neutral red and crystal violet Biol Pharm Bull 1996 19 1518 1520 10.1248/bpb.19.1518 8951178

[B39] Irizarry RA Hobbs B Collin F Beazer-Barclay YD Antonellis KJ Scherf U Speed TP Exploration, normalization, and summaries of high density oligonucleotide array probe level data Biostatistics 2003 4 249 264 10.1093/biostatistics/4.2.249 12925520

[B40] Bolstad BM Irizarry RA Astrand M Speed TP A comparison of normalization methods for high density oligonucleotide array data based on variance and bias Bioinformatics 2003 19 185 193 10.1093/bioinformatics/19.2.185 12538238

[B41] Edgar R Domrachev M Lash AE Gene Expression Omnibus: NCBI gene expression and hybridization array data repository Nucleic Acids Res 2002 30 207 210 10.1093/nar/30.1.207 11752295 PMC99122

[B42] Smyth GK Michaud J Scott HS Use of within-array replicate spots for assessing differential expression in microarray experiments Bioinformatics 2005 21 2067 2075 10.1093/bioinformatics/bti270 15657102

[B43] Benjamini Y Hochberg Y Controlling the false discovery rate: a practical and powerful approach to multiple testing J Royal Statistical Soc Series B (Methodological) 1995 57 289 300

[B44] Saeed AI Bhagabati NK Braisted JC Liang W Sharov V Howe EA Li J Thiagarajan M White JA Quackenbush J TM4 microarray software suite Methods Enzymol 2006 411 134 193 16939790 10.1016/S0076-6879(06)11009-5

[B45] Brock G Datta S Pihur V Datta S clValid: an R package for cluster validation J Stat Softw 2008 25 1 22

[B46] Huang dW Sherman BT Lempicki RA Systematic and integrative analysis of large gene lists using DAVID bioinformatics resources Nat Protoc 2009 4 44 57 19131956 10.1038/nprot.2008.211

[B47] Franco MI Turin L Mershin A Skoulakis EM Molecular vibration-sensing component in Drosophila melanogaster olfaction Proc Natl Acad Sci USA 2011 108 3797 3802 10.1073/pnas.1012293108 21321219 PMC3048096

[B48] Tran AH Berger A Wu GE Kee BL Paige CJ Early B-cell factor regulates the expression of Hemokinin-1 in the olfactory epithelium and differentiating B lymphocytes J Neuroimmunol 2011 232 41 50 10.1016/j.jneuroim.2010.09.027 20965576

[B49] Cunin P Caillon A Corvaisier M Garo E Scotet M Blanchard S Delneste Y Jeannin P The tachykinins substance P and hemokinin-1 favor the generation of human memory Th17 cells by inducing IL-1beta, IL-23, and TNF-like 1A expression by monocytes J Immunol 2011 186 4175 4182 10.4049/jimmunol.1002535 21368235

[B50] Madaan V Wilson DR Neuropeptides: relevance in treatment of depression and anxiety disorders Drug News Perspect 2009 22 319 324 10.1358/dnp.2009.22.6.1395255 19771321

[B51] Alherbish A Charrois TL Ackman ML Tsuyuki RT Ezekowitz JA The prevalence of natural health product use in patients with acute cardiovascular disease PLoS ONE 2011 6 e19623 10.1371/journal.pone.0019623 21573067 PMC3090400

[B52] Barbancey J Pratique Homéopathique en psycho-pathologie, Tome II 1987 Paris: Editions Similia

[B53] Danno K Colas A Masson JL Bordet MF Homeopathic treatment of migraine in children: results of a prospective, multicenter, observational study J Altern Complement Med 2012 19 119 123 22978244 10.1089/acm.2011.0821

[B54] Paris A Gonnet N Chaussard C Belon P Rocourt F Saragaglia D Cracowski JL Effect of homeopathy on analgesic intake following knee ligament reconstruction: a phase III monocentre randomized placebo controlled study Br J Clin Pharmacol 2008 65 180 187 10.1111/j.1365-2125.2007.03008.x 18251757 PMC2291233

[B55] Bellavite P Conforti A Piasere V Ortolani R Immunology and homeopathy. 1. Historical background eCAM 2005 2 441 452 16322800 10.1093/ecam/neh141 PMC1297514

[B56] Shang A Huwiler-Müntener K Nartey L Jüni P Dörig S Sterne JAC Pewsner D Egger M Are the clinical effects of homoeopathy placebo effects? Comparative study of placebo-controlled trials of homoeopathy and allopathy Lancet 2005 366 726 732 10.1016/S0140-6736(05)67177-2 16125589

[B57] Jonas WB Kaptchuk TJ Linde K A critical overview of homeopathy Ann Intern Med 2003 138 393 399 10.7326/0003-4819-138-5-200303040-00009 12614092

[B58] Calabrese EJ Jonas WB Evaluating homeopathic drugs within a biomedical framework Hum Exp Toxicol 2010 29 545 549 10.1177/0960327110369775 20558604

[B59] Plant KE Anderson E Simecek N Brown R Forster S Spinks J Toms N Gibson GG Lyon J Plant N The neuroprotective action of the mood stabilizing drugs lithium chloride and sodium valproate is mediated through the up-regulation of the homeodomain protein Six1 Toxicol Appl Pharmacol 2009 235 124 134 10.1016/j.taap.2008.10.019 19101580

[B60] Lai CK Chan YW Confirmation of Gelsemium poisoning by targeted analysis of toxic Gelsemium alkaloids in urine J Anal Toxicol 2009 33 56 61 10.1093/jat/33.1.56 19161670

[B61] Calabrese EJ Jonas WB Homeopathy: clarifying its relationship to hormesis Hum Exp Toxicol 2010 29 531 536 10.1177/0960327110369857 20558601

[B62] Iavicoli I Calabrese EJ Nascarella MA Exposure to nanoparticles and hormesis Dose Response 2010 8 501 517 10.2203/dose-response.10-016.Iavicoli 21191487 PMC2990066

[B63] Van Wijk R Wiegant FA Postconditioning hormesis and the homeopathic Similia principle: molecular aspects Hum Exp Toxicol 2010 29 561 565 10.1177/0960327110369860 20558607

[B64] Bell IR Schwartz GE Adaptive network nanomedicine: an integrated model for homeopathic medicine Front Biosci (Schol Ed) 2013 5 685 708 23277079 10.2741/s400

[B65] Venard C Boujedaini N Belon P Mensah-Nyagan AG Patte-Mensah C Regulation of neurosteroid allopregnanolone biosynthesis in the rat spinal cord by glycine and the alkaloidal analogs strychnine and gelsemine Neuroscience 2008 153 154 161 10.1016/j.neuroscience.2008.02.009 18367344

[B66] Bellavite P Conforti A Pontarollo F Ortolani R Immunology and homeopathy. 2. Cells of the immune system and inflammation eCAM 2006 3 13 24 16550219 10.1093/ecam/nek018 PMC1375241

[B67] Witt CM Bluth M Albrecht H Weisshuhn TE Baumgartner S Willich SN The in vitro evidence for an effect of high homeopathic potencies–a systematic review of the literature Complement Ther Med 2007 15 128 138 10.1016/j.ctim.2007.01.011 17544864

[B68] Sainte-Laudy J Belon P Inhibition of basophil activation by histamine: a sensitive and reproducible model for the study of the biological activity of high dilutions Homeopathy 2009 98 186 197 10.1016/j.homp.2009.09.009 19945674

[B69] Majewsky V Arlt S Shah D Scherr C Jager T Betti L Trebbi G Bonamin L Klocke P Baumgartner S Use of homeopathic preparations in experimental studies with healthy plants Homeopathy 2009 98 228 243 10.1016/j.homp.2009.09.012 19945677

[B70] Bellavite P Marzotto M Olioso D Moratti E Conforti A High-dilution effects revisited. 1. Physicochemical aspects Homeopathy 2014 103 4 21 10.1016/j.homp.2013.08.003 24439452

[B71] Anick DJ Ives JA The silica hypothesis for homeopathy: physical chemistry Homeopathy 2007 96 189 195 10.1016/j.homp.2007.03.005 17678816

[B72] Chikramane PS Suresh AK Bellare JR Kane SG Extreme homeopathic dilutions retain starting materials: A nanoparticulate perspective Homeopathy 2010 99 231 242 10.1016/j.homp.2010.05.006 20970092

[B73] Relaix S Leheny RL Reven L Sutton M Memory effect in composites of liquid crystal and silica aerosil Phys Rev E Stat Nonlin Soft Matter Phys 2011 84 061705 22304107 10.1103/PhysRevE.84.061705

[B74] Baier G Costa C Zeller A Baumann D Sayer C Araujo PH Mailänder V Musyanovych A Landfester K BSA adsorption on differently charged polystyrene nanoparticles using isothermal titration calorimetry and the influence on cellular uptake Macromol Biosci 2011 11 628 638 10.1002/mabi.201000395 21384550

[B75] Bel HS Magnin A Petrier C Boufi S Starch nanoparticles formation via high power ultrasonication Carbohydr Polym 2013 92 1625 1632 10.1016/j.carbpol.2012.11.022 23399199

[B76] Upadhyay RP Nayak C Homeopathy emerging as nano medicine Int J High Dilution Res 2011 10 299 310

[B77] Das S Das J Samadder A Bhattacharyya SS Das D Khuda-Bukhsh AR Biosynthesized silver nanoparticles by ethanolic extracts of Phytolacca decandra, Gelsemium sempervirens, Hydrastis canadensis and Thuja occidentalis induce differential cytotoxicity through G2/M arrest in A375 cells Colloids Surf B Biointerfaces 2013 101 325 336 23010037 10.1016/j.colsurfb.2012.07.008

[B78] Prakash DJ Arulkumar S Sabesan M Effect of nanohypericum (Hypericum perforatum gold nanoparticles) treatment on restraint stressinduced behavioral and biochemical alteration in male albino mice Pharmacognosy Res 2010 2 330 334 10.4103/0974-8490.75450 21713134 PMC3111690

[B79] Bhattacharyya SS Paul S Khuda-Bukhsh AR Encapsulated plant extract (Gelsemium sempervirens) poly (lactide-co-glycolide) nanoparticles enhance cellular uptake and increase bioactivity in vitro Exp Biol Med (Maywood ) 2010 235 678 688 10.1258/ebm.2010.009338 20511672

[B80] Schwartz GE Russek LG Bell IR Riley D Plausibility of homeopathy and conventional chemical therapy: the systemic memory resonance hypothesis Med Hypotheses 2000 54 634 637 10.1054/mehy.1999.0913 10859655

[B81] Bellavite P Complexity science and homeopathy. A synthetic overview Homeopathy 2003 92 203 212 10.1016/j.homp.2003.08.002 14587687

[B82] Bellavite P Olioso D Marzotto M Moratti E Conforti A A dynamic network model of the similia principle Complement Ther Med 2013 21 750 761 10.1016/j.ctim.2013.09.001 24280484

[B83] Bellavite P Marzotto M Olioso D Moratti E Conforti A High-dilution effects revisited. 2. Pharmacodynamic mechanisms Homeopathy 2014 103 22 43 10.1016/j.homp.2013.08.002 24439453

[B84] Ramo P Kesseli J Yli-Harja O Perturbation avalanches and criticality in gene regulatory networks J Theor Biol 2006 242 164 170 10.1016/j.jtbi.2006.02.011 16574157

[B85] Likhoshvai VA Fadeev SI Kogai VV Khlebodarova TM On the chaos in gene networks J Bioinform Comput Biol 2013 11 1340009 10.1142/S021972001340009X 23427991

[B86] Morita K Tsumoto K Aihara K Possible effects of depolarizing GABAA conductance on the neuronal input–output relationship: a modeling study J Neurophysiol 2005 93 3504 3523 10.1152/jn.00988.2004 15689391

[B87] Gupta K Singh HP Biswal B Ramaswamy R Adaptive targeting of chaotic response in periodically stimulated neural systems Chaos 2006 16 023116 10.1063/1.2204749 16822019

[B88] Qi Y Watts AL Kim JW Robinson PA Firing patterns in a conductance-based neuron model: bifurcation, phase diagram, and chaos Biol Cybern 2013 107 15 24 10.1007/s00422-012-0520-8 22990669

[B89] Szolnoki Z A dynamically changing intracellular water network serves as a universal regulator of the cell: the water-governed cycle Biochem Biophys Res Commun 2007 357 331 334 10.1016/j.bbrc.2007.03.161 17420007

