# Supplementary figures and images for: Extreme sensitivity of gene expression in human SH-SY5Y neurocytes to ultra-low doses of Gelsemium sempervirens
Source: BMC Complement Altern Med. 2014 Mar 19;14:104. doi: 10.1186/1472-6882-14-104 (PMC3999908; doi:10.1186/1472-6882-14-104)

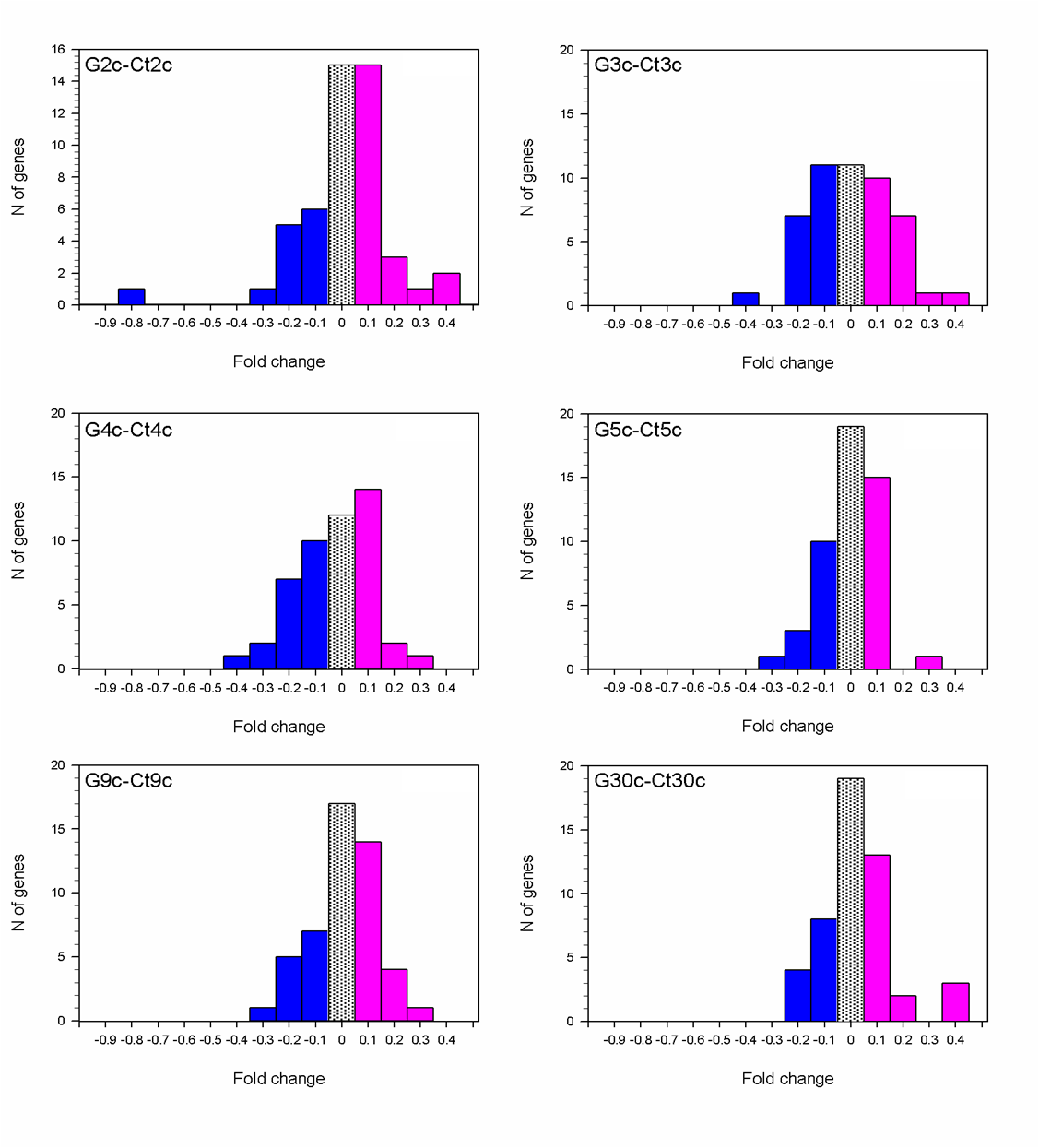

Supplement: Additional file 2 — Frequency of fold change values in a randomly chosen gene-set after Gelsemium s. treatments. A list of 49 genes was generated by randomized selection from the whole transcriptome using SPSS software (excluding the differentially expressed genes) and fold change was calculated from the difference of mean Log2 fluorescence values of Gelsemium s.-treated samples (Gnc) vs those of controls (Ctnc). Absolute fold changes less than or equal to 0.05 were considered null. Blue bars: frequencies of genes with negative fold change (< -0.05); grey bars: frequency of unaffected genes (from -0.05 to 0.05); pink bars: frequencies of genes with positive fold change (> 0.05). For these randomly selected genes, Fisher exact test is not significant in any dilution. [file 1472-6882-14-104-S2.tiff]
